# Intratumoral nanobody–IL-2 fusions that bind the tumor extracellular matrix suppress solid tumor growth in mice

**DOI:** 10.1093/pnasnexus/pgac244

**Published:** 2022-11-03

**Authors:** Emi A Lutz, Noor Jailkhani, Noor Momin, Ying Huang, Allison Sheen, Byong H Kang, K Dane Wittrup, Richard O Hynes

**Affiliations:** Koch Institute for Integrative Cancer Research, Massachusetts Institute of Technology, Cambridge, MA 02139, USA; Department of Biological Engineering, Massachusetts Institute of Technology, Cambridge, MA 02139, USA; Koch Institute for Integrative Cancer Research, Massachusetts Institute of Technology, Cambridge, MA 02139, USA; Koch Institute for Integrative Cancer Research, Massachusetts Institute of Technology, Cambridge, MA 02139, USA; Department of Biological Engineering, Massachusetts Institute of Technology, Cambridge, MA 02139, USA; Koch Institute for Integrative Cancer Research, Massachusetts Institute of Technology, Cambridge, MA 02139, USA; Koch Institute for Integrative Cancer Research, Massachusetts Institute of Technology, Cambridge, MA 02139, USA; Department of Biological Engineering, Massachusetts Institute of Technology, Cambridge, MA 02139, USA; Koch Institute for Integrative Cancer Research, Massachusetts Institute of Technology, Cambridge, MA 02139, USA; Department of Biological Engineering, Massachusetts Institute of Technology, Cambridge, MA 02139, USA; Koch Institute for Integrative Cancer Research, Massachusetts Institute of Technology, Cambridge, MA 02139, USA; Department of Biological Engineering, Massachusetts Institute of Technology, Cambridge, MA 02139, USA; Department of Chemical Engineering, Massachusetts Institute of Technology, Cambridge, MA 02139, USA; Koch Institute for Integrative Cancer Research, Massachusetts Institute of Technology, Cambridge, MA 02139, USA; Department of Biology, Massachusetts Institute of Technology, Cambridge, MA 02139, USA

**Keywords:** nanobody, cytokine, cancer

## Abstract

Confining cytokine exposure to the tumors would greatly enhance cancer immunotherapy safety and efficacy. Immunocytokines, cytokines fused to tumor-targeting antibodies, have been developed with this intention, but without significant clinical success to date. A critical limitation is uptake by receptor-expressing cells in the blood, that decreases the dose at the tumor and engenders toxicity. Small-format immunocytokines, constructed with antibody fragments, are hypothesized to improve tumor specificity due to rapid systemic clearance. However, effective design criteria for small-format immunocytokines need further examination. Here, we engineer small interleukin-2 (IL-2) immunocytokines fused to nanobodies with nanomolar to picomolar affinities for the tumor-specific EIIIB domain of fibronectin (also known as EDB). Upon intravenous delivery into immunocompetent mice, such immunocytokines led to similar tumor growth delay as size-matched untargeted IL-2. Intratumoral (i.t.) delivery imparted improved survival dependent on affinity to EIIIB. I.t. administration offers a promising avenue to deliver small-format immunocytokines, given effective affinity for the tumor microenvironment.

Significance Statement Cytokines like interleukin-2 (IL-2) are promising cancer therapeutics. Fusing cytokines to a tumor-specific binding moiety is one potential strategy to improve cytokine efficacy and toxicity, but it is unclear to what extent IL-2 can be redirected to the tumor. In this work, we develop nanobody–IL-2 fusions that are specific for EIIIB, a component of the tumor extracellular matrix. After intravenous administration into tumor-bearing mice, EIIIB-specific nanobody–IL-2 fusions led to similar delay in tumor growth compared with size-matched untargeted IL-2. In contrast, following intratumoral administration, EIIIB-specific nanobody–IL-2 fusions were able to cure tumors in a nanobody-dependent manner. These experiments help clarify principles for engineering and administration of tumor extracellular matrix specific nanobody–cytokine fusions.

## Introduction

Cytokines can potently activate antitumor immune cells, and so are promising cancer immunotherapies. In the 1990s, interleukin-2 (IL-2) earned FDA-approval after achieving complete responses in a small subset of patients with metastatic renal cell carcinoma and metastatic melanoma (1, 2). Unfortunately, the majority of treated patients also experienced severe, and in some cases fatal, adverse reactions to treatment (3). Toxicity from IL-2 treatment arises from on-target, off-tumor activation of systemically circulating immune cells. Strategies to improve IL-2 therapy depend on preferentially increasing tumor exposure while decreasing systemic exposure.

Intravenous administration of cytokines fused to tumor-targeting vehicles aims to direct their immunomodulatory effects to the tumor. Immunocytokines, with cytokines fused to antibodies or antibody fragments specific to tumor-associated antigens, have been tested to this end with some promise. However, recent clinical trials show that dose-limiting side effects and low efficacy are not fully ameliorated by such attempts at tumor targeting (4). Previously, we demonstrated in mice that large antibody–IL-2 fusions have compromised tumor-targeting due to their prolonged circulation and uptake by abundant IL-2-receptor-expressing cells in the blood (5). Tzeng et al. (5) predicted that smaller immunocytokines may, for some combinations of parameters, achieve higher tumor to blood ratios, because of their rapid systemic clearance.

Here, we explore small-format IL-2 immunocytokines with affinity to the tumor extracellular matrix, a major component of the tumor microenvironment, rather than cell-surface antigens of tumor cells. Specifically, the alternatively spliced EIIIB (EDB) domain of fibronectin (FN), which is not expressed in healthy adult tissue, is widely expressed in a variety of cancers as a component of the tumor extracellular matrix (6). EIIIB is a promising translational target, with an identical amino acid sequence in humans and mice. EIIIB+FN has been pursued by others in the context of IL-2 (7), as well as other cytokines and antitumor therapies (8). As one example, Jailkhani et al. generated the nanobody NJB2, which binds EIIIB with 2 nM affinity and has been applied in tumor imaging (9) and chimeric antigen receptor (CAR) T cell therapy (10). Here, we use yeast surface display to affinity mature NJB2 and engineer novel nanobodies that bind EIIIB with picomolar affinity, and demonstrate the necessity for such high affinity for efficacy following intratumoral (i.t.) administration.

In this work, we develop and test small (32 kDa) IL-2 immunocytokines with a range of affinities to EIIIB in immunocompetent mice bearing B16F10 or 4T1 tumors. When delivered intravenously, even picomolar affinity to EIIIB did not produce substantially improved efficacy or altered binding to immune cell types compared to size-matched, untargeted IL-2. However, after local i.t. administration, anti-EIIIB IL-2 fusions enabled a high B16F10 melanoma cure rate that was not observed with untargeted IL-2. We demonstrate here that immunocytokines originally designed for systemic delivery can alternatively provide a powerful modality for extended retention following i.t. injection, provided that their target-binding affinity is sufficiently high. In the optimal implementation, this approach ablates established solid tumors and primes protective adaptive immunity.

## Results

### Nanobody–IL-2 immunocytokine with nanomolar affinity to the EIIIB domain yield efficacy similar to untargeted IL-2 after intravenous administration

**Fig. 1.**
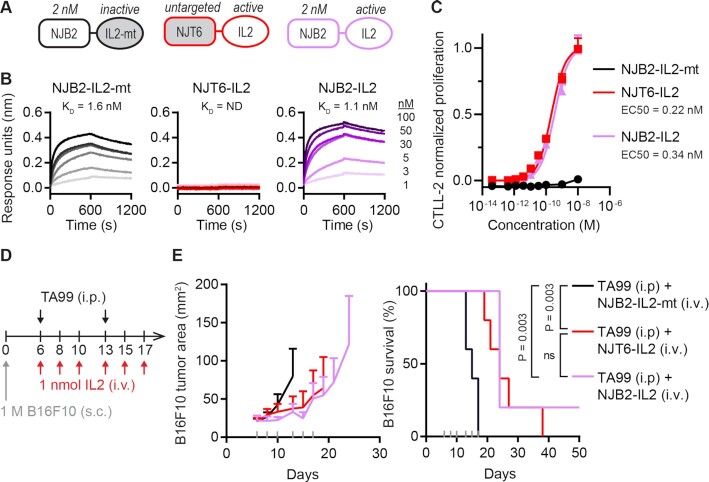
IL-2 immunocytokine with nanomolar affinity to EIIIB yields similar efficacy to untargeted IL-2 in B16F10 tumors. (A) Schematic of IL-2 fusions with the nanobody as a rectangle and the IL-2 as an oval. The nanobody and IL-2 are separated by a glycine–serine (G_5_S) linker, and the entire protein is ∼32 kDa. (B) Association and dissociation curves (600 seconds each) are shown for indicated IL-2 fusions as measured by BLI using streptavidin tips coated with biotinylated EIIIB. Dark to light indicates analyte concentration of 100, 50, 30, 5, 3, and 1 nM. ND, not determined. (C) Dose-dependent, normalized CTLL-2 cell proliferation in response to IL-2 fusion proteins (mean + SD; *n* = 3). (D) B16F10 study timeline. Mice were inoculated with 1 M B16F10 cells subcutaneously (s.c.) in the right flank on day 0. Mice were treated on days indicated with 100 μg TA99 (i.p.) and 1 nmol (32 μg) IL-2 fusions (i.v.). (E) Tumor growth (left) and survival (right). Gray ticks above the *x*-axis mark treatment days. Tumor area (mean + SD) is shown until a mouse in that group is euthanized. Statistical significance for survival was generated by a log-rank Mantel–Cox test. ns, not significant. *n* = 5 for all groups.

To test whether nanobodies specific to the EIIIB domain of FN can be used for targeted delivery and persistence of cytokines in the tumor ECM, we expressed murine IL-2 fused to the NJB2 nanobody, previously shown to bind EIIIB with 2 nM affinity (9). As a size-matched control, we also constructed a fusion of IL-2 with the NJT6 nanobody, which has no murine target (9) (Fig. 1A). As an inactive control, we fused NJB2 to murine IL-2 bearing point mutations described to abrogate cytokine activity (NJB2-IL2-mt; Figure S1A) (11, 12). All recombinant immunocytokines were ∼32 kDa (Figure S1B and C). Consistent with this small size, all constructs had rapid systemic clearance, and less than 0.3% of the injected dose remained in the blood 24 hours after retro-orbital injection (Figure S1D). Both NJB2-IL2 and NJB2-IL2-mt retained their single-digit nanomolar affinity to EIIIB, whereas NJT6-IL2 had no measurable affinity (Fig. 1B). The two IL-2 fusions had identical bioactivity on CTLL-2 cells, a murine T cell line responsive to IL-2, while the IL2-mt fusion did not induce CTLL-2 proliferation (Fig. 1C).

To investigate the therapeutic efficacy of the nanobody–IL-2 fusions, we employed the B16F10 melanoma model, previously shown to express EIIIB+FN by microscopy, immuno-PET/CT-imaging, and immunohistochemistry (9). Since the B16F10 model is immunologically cold and unresponsive to most single-agent immunotherapies, we combined our immunocytokine treatment with TA99, a murine IgG2a tumor-targeting antibody against tumor-associated antigen tyrosinase-related protein-1. TA99 has been shown to activate natural killer (NK) cells and prime CD8^+^ T cells that depend on IL-2 for antitumor activity (13). Mice bearing subcutaneous B16F10 tumors were treated with the nanobody–IL-2 fusions intravenously (i.v.), and with TA99 intraperitoneally (i.p.; Fig. 1D). Compared to the inactive control NJB2-IL2-mt, both NJT6-IL2 and NJB2-IL2 improved survival of treated mice (*P* = 0.003; Fig. 1E). However, despite its 2 nM affinity for EIIIB, NJB2-IL2 did not improve survival compared to the untargeted size-matched control, NJT6-IL2, using this regimen for i.v. administration (*P* = 0.40). Of note, 1 nmol dose into an approximately 2 mL blood volume amounts to a postinjection blood concentration of ∼500 nM, two orders of magnitude above the K_D_ of NJB2.

Given the result that NJB2-IL2 (2 nM affinity to EIIIB) and NJT6-IL2 (no affinity to EIIIB) elicit similar, modest delay in B16F10 tumor outgrowth after i.v. administration, we sought to understand better the design criteria for effective IL-2 immunocytokines. We hypothesized that three key parameters may impact the efficacy of EIIIB-targeted small IL-2 immunocytokines: (1) nanobody affinity to EIIIB, (2) abundance of EIIIB at the tumor site, and (3) route of administration. We moved on to study each of these parameters to uncover how best to design and dose small-format IL-2 immunocytokines.

### Engineered LMJ1.2C and LMJ2.5I nanobodies bind EIIIB with picomolar affinity

**Fig. 2.**
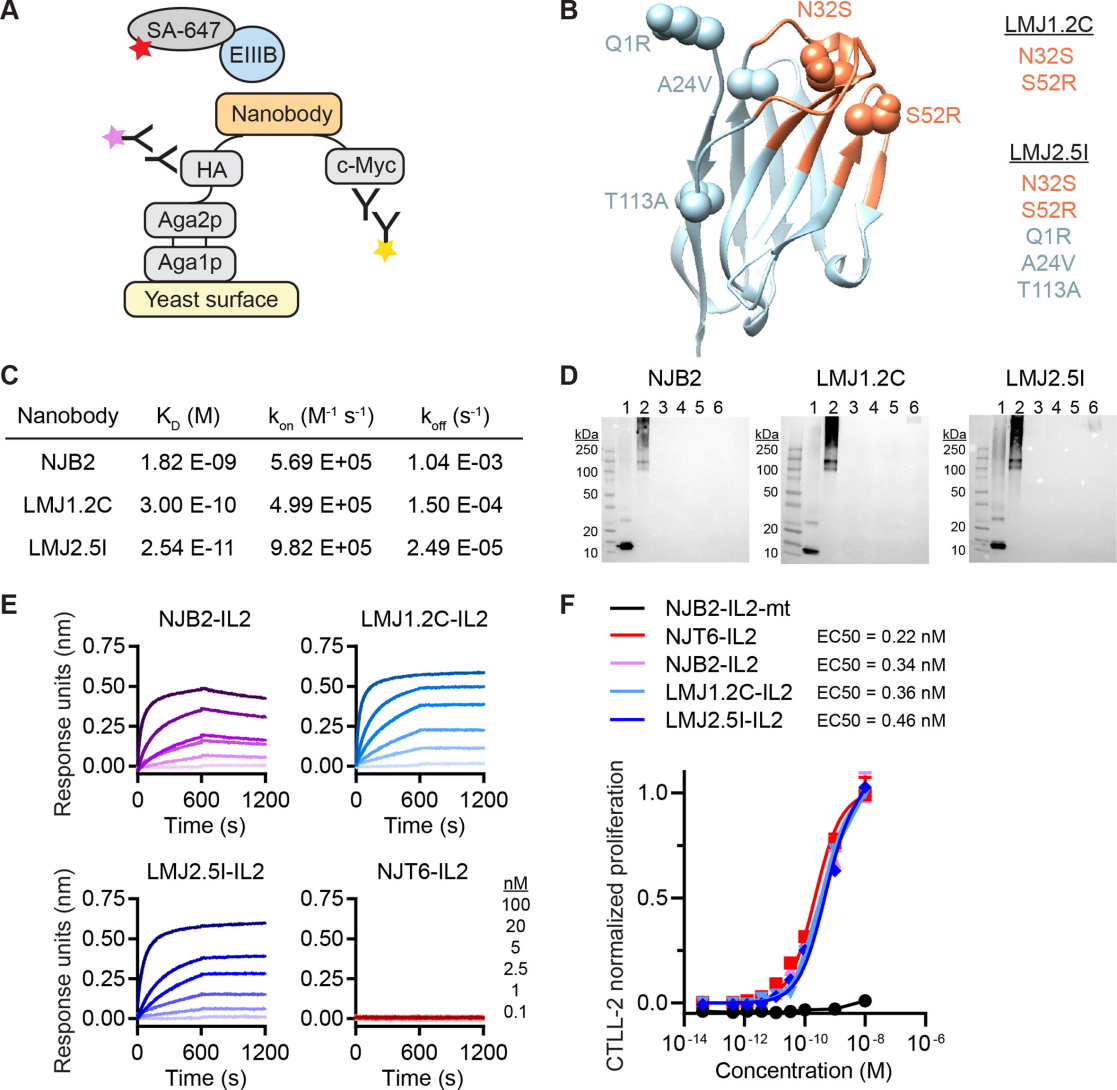
Engineering nanobodies with picomolar affinity to EIIIB via yeast surface display. (A) Schematic showing how nanobody libraries were expressed on the surface of yeast as an Aga2 fusion. Nanobody expression is detected by fluorescent antibodies that bind to epitope tags hemagglutinin (HA) or c-Myc. Yeast were selected for binding to biotinylated EIIIB, as detected by Streptavidin Alexa Fluor 647 (SA-647). (B) A homology model of NJB2 was generated using ABodyBuilder on the SAbPred server (15–17). CDRs (orange) and framework (blue) are shown in ribbon format. The location of mutations in LMJ1.2C and LMJ2.5I are shown in sphere format. (C) Biotin-tagged nanobodies were analyzed by BLI. Equilibrium dissociation constant (K_D_), rate of association (k_on_), and rate of dissociation (k_off_) using 1:1 curve fits are reported with curves shown in Figure S3. (D) Biotin-tagged nanobodies were analyzed by immunoblot. Lanes 1 to 6, (1) EIIIB-His6, (2) FN 7–15 EIIIB, (3) human plasma FN, (4) mouse plasma FN, (5) normal murine lung ECM, and (6) His-GFP protein. (E) Association and dissociation curves (600 seconds each) are shown for indicated IL-2 fusions as measured by BLI using streptavidin tips coated with biotinylated EIIIB. Dark to light indicates analyte concentration of 100, 20, 5, 2.5, 1, and 0.1 nM. (F) Dose-dependent, normalized CTLL-2 cell proliferation in response to IL-2 fusion proteins (mean + SD; *n* = 3).

The fraction of target bound by a drug depends on the concentrations of drug and target, along with the equilibrium dissociation constant (K_D_). Thus, affinity might be expected to play a significant role in therapeutic outcome (although note that in the experiments shown in Fig. 1, EIIIB binding does not appear to improve efficacy relative to a nonbinding nanobody fusion). To assess whether higher affinity versions of NJB2 would improve therapeutic benefit, we engineered tighter binders to EIIIB using yeast surface display (14) (Fig. 2A and Figure S2A and B). In brief, we generated a yeast library displaying NJB2 mutants and sorted clones with tighter binding to EIIIB. A pool of three of the most promising clones served as template for a second-round yeast library mutagenesis that we stringently screened for further enhancements in affinity. From these screens, we identified 10 nanobodies of interest with frequently observed mutations in both their complementarity-determining regions (CDR) and framework regions (Figure S2C). The 10 nanobodies were recombinantly expressed and purified with C-terminal polyhistidine tags. All nanobodies demonstrated subnanomolar affinity to EIIIB as measured by biolayer interferometry (BLI; Figure S3). We proceeded with clones LMJ1.2C and LMJ2.5I, which respectively contain two and five mutations compared with the parental nanobody NJB2, as visualized with a homology model of NJB2 (Fig. 2B) (15–17). As measured by BLI, LMJ1.2C demonstrated a 300-pM K_D_ and LMJ2.5I demonstrated a 25-pM K_D_ (Fig. 2C and Figure S3). To test their specificity for EIIIB+FN, the nanobodies were tagged at the C-terminus with biotin via sortase-mediated tagging and tested by immunoblotting. All engineered nanobodies showed specificity of binding to fragments that contained EIIIB (EIIIB-His and FN (7-15) EIIIB), while no binding was observed for negative controls including human and mouse plasma FNs, ECM enriched from normal murine lung or His-GFP protein (Fig. 2D and Figure S4). We then generated recombinant LMJ1.2C and LMJ2.5I fused to IL-2 (Figure S5A and B). LMJ1.2C-IL2 and LMJ2.5I-IL2 maintained their high affinity for EIIIB, as demonstrated by BLI (Fig. 2E and Figure S3B) and ELISA (Figure S5C), as well as IL-2 functional activity on CTLL-2 cells (Fig. 2F).

### Nanomolar and picomolar IL-2 immunocytokines lead to mild tumor growth delay after intravenous administration

**Fig. 3.**
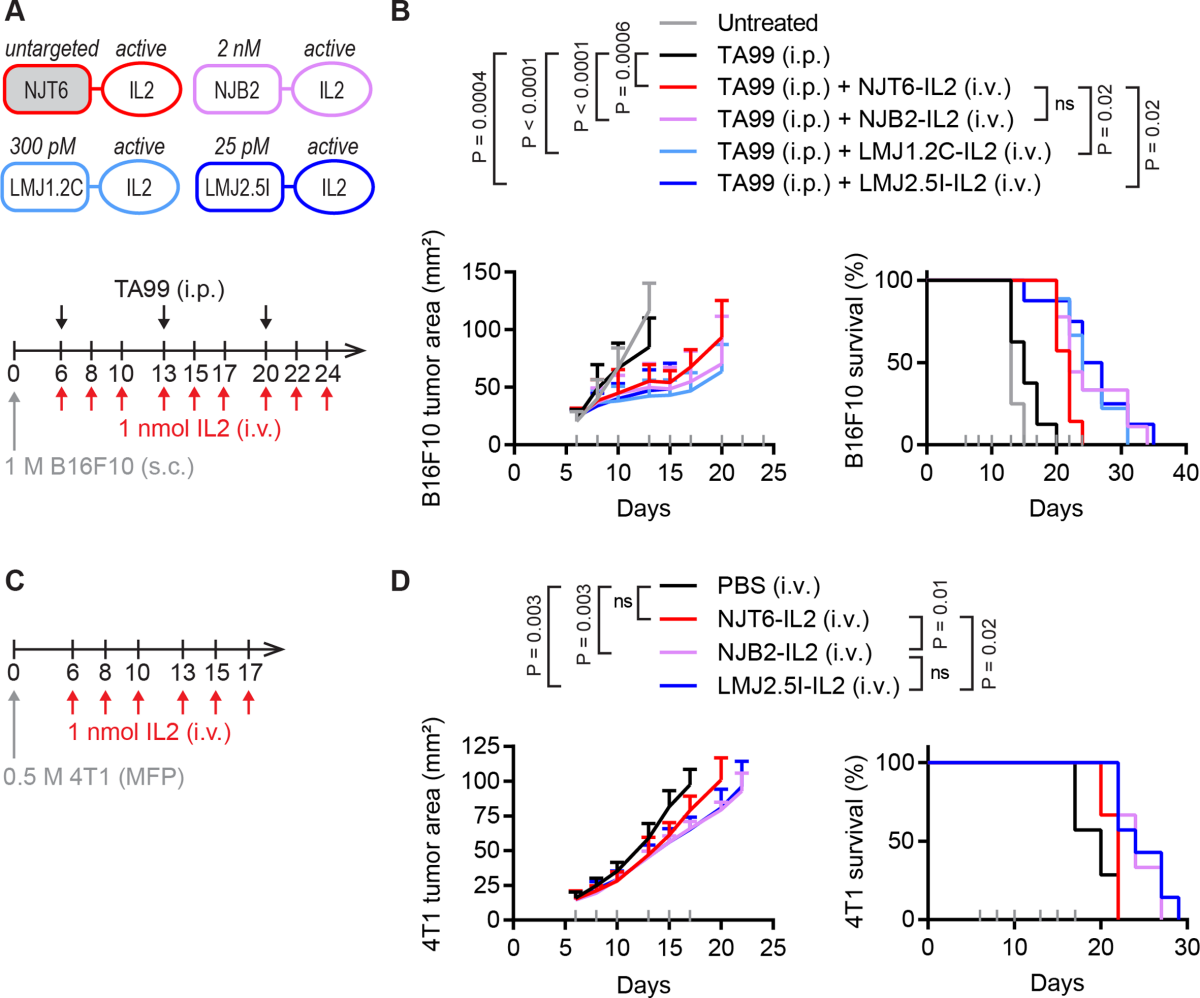
EIIIB-specific IL-2 immunocytokines lead to modest survival extension after intravenous administration. (A) Schematic of protein fusions (top) and B16F10 study timeline (bottom). Mice were inoculated with 1 M B16F10 cells subcutaneously (s.c.) in the right flank on day 0. Mice were treated on days indicated with 100 μg TA99 (i.p.) and 1 nmol (32 μg) IL-2 fusions (i.v.). (B) Tumor growth (left) and survival (right). Gray ticks above the *x*-axis mark treatment days. Tumor area (mean + SD) is shown until a mouse in that group is euthanized. Survival comparisons were generated by a log-rank Mantel–Cox test. ns, not significant. *n* = 7∼9. (C) 4T1 study timeline. Mice were inoculated with 0.5 M 4T1 cells in the mammary fat pad (MFP) on day 0 and treated on days indicated with 1 nmol (32 μg) IL-2 fusions (i.v.). (D) Tumor growth (left) and survival (right). Gray ticks above the *x*-axis mark treatment days. Tumor area (mean + SD) is shown until a mouse in that group is euthanized. Survival comparisons were generated by a log-rank Mantel–Cox test. ns, not significant. *n* = 6∼7.

With novel picomolar nanobodies in hand, we again tested how affinity to EIIIB affects efficacy after i.v. treatment of IL-2. Mice bearing B16F10 tumors were treated with IL-2 fusions (i.v.) and TA99 (i.p.), this time for 3 weeks of treatment (Fig. 3A). Compared to the TA99-only control, the addition of any nanobody–IL-2 fusion improved survival (Fig. 3B). Compared to the untargeted NJT6-IL2 control, NJB2-IL2 with nanomolar affinity to EIIIB led to nonsignificant trends toward survival extension (*P* = 0.10), while LMJ1.2C-IL2 and LMJ2.5I-IL2 with picomolar affinity to EIIIB led to modest extension of survival (*P* = 0.02 for both).

To test whether we would see better outcomes in tumors that express higher levels of EIIIB + FN, we also tested the anti-EIIIB IL-2 fusions i.v. as a monotherapy in the 4T1 orthotopic breast cancer model, which has been shown to express more EIIIB than the B16F10 model (9) (Fig. 3C). In this setting, we found that both the nanomolar binder NJB2-IL2 and the picomolar binder LMJ2.5I-IL2 extended survival compared to the untargeted NJT6-IL2 (*P* = 0.01 for NJB2-IL2 and *P* = 0.02 for LMJ2.5I-IL2; Fig. 3D). However, the benefit was modest, and all mice succumbed to disease by day 29. No weight loss was observed in treated mice in either the B16F10 or the 4T1 study (Figure S6A and B).

### EIIIB-specific IL-2 immunocytokines have similar cellular biodistribution after intravenous administration

**Fig. 4.**
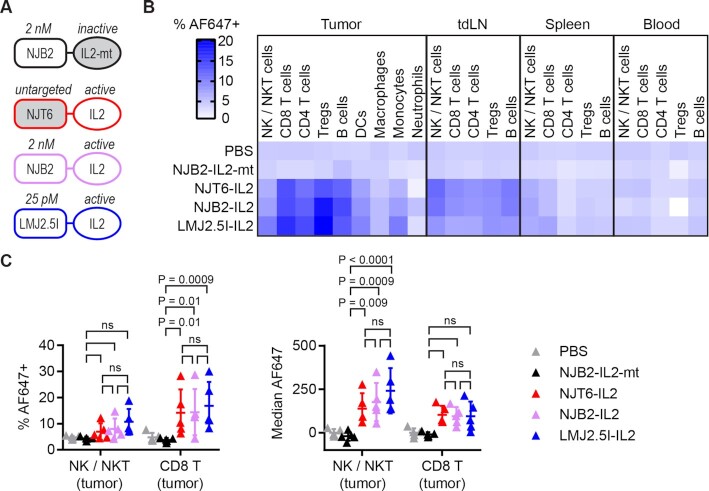
Targeted and untargeted IL-2 have similar cellular biodistribution after intravenous administration. Mice were inoculated with 1 M B16F10 cells s.c. in the right flank on day 0. Mice were treated i.v. on day 8 with 1 nmol (32 μg) AF647-labeled IL-2 fusions. A total of 24 hours later, mice were sacrificed for necropsy and flow cytometry. (A) Schematic of IL-2 fusions. (B) Heat map displaying % AF647^+^ in indicated cell types and organs (mean of *n* = 5). Individual data points and statistics reported in Figure S8. (C) % AF647^+^ (left) and median AF647 (right) for NK/NKT cells and CD8^+^ T cells in the tumor (mean ± SD; *n* = 5). When reporting median AF647, background levels from PBS-treated mice were subtracted. Data were analyzed with two-way analysis of variance (ANOVA) with Tukey’s multiple comparisons test.

Intravenously delivered IL-2 with picomolar affinity to EIIIB had only a modest impact on tumor growth compared to untargeted IL-2 in two different tumor models (Fig. 3).We have previously showed that the biodistribution of large format IL-2 immunocytokines is dominated by the IL-2 moiety (5). When delivered systemically, large format immunocytokines bind to cells expressing IL-2 receptor in the systemic circulation, the “systemic sink.” To test how the nanobody and cytokine moieties impact cellular biodistribution of small-format nanobody–IL-2 fusions, we assessed drug uptake in different immune cells across multiple organs. We compared active IL-2 fused to NJT6, NJB2, or LMJ2.5I, as well as NJB2 fused to size-matched inactive mutant IL-2 (Fig. 4A). The nanobody–IL-2 fusions were fluorescently labeled with Alexa Fluor 647 (AF647) and administered i.v. into mice bearing 8-day-old B16F10 tumors. A total of 24 hours later, we harvested tumors, tumor draining lymph nodes (tdLNs), spleens, and blood, and studied the biodistribution of the immunocytokines in immune cells via flow cytometry (Figure S7).

The percentage of each cell population positive for AF647 is shown as a heatmap (Fig. 4B), with all data and statistics in Figure S8. There was minimal signal in mice treated with PBS or inactive IL-2, as expected, while the three proteins with active IL-2 had similar levels of drug uptake in different immune cell populations across the tested organs. Unlike what has been observed for the large format immunocytokines, these small-format nanobody–IL-2 fusions had minimal signal in spleen and blood, suggesting rapid clearance from systemic circulation. We have previously seen that CD8^+^ T cells and NK cells in the tumor are especially important in the uptake and efficacy of larger IL-2 constructs in combination with TA99 (5, 13, 18). Regardless of affinity to EIIIB, the three active IL-2 fusions had similar impacts on these populations, namely increased % AF647^+^ for CD8^+^ T cells in the tumor, increased median AF647 for NK/NKT cells in the tumor (Fig. 4C), and no significant differences in cell counts (Figure S8E).

These data on the systemic distribution of the nanobody–IL-2 fusions are consistent with the observed mild extensions of survival. The NJT6-IL2 fusion that lacks any affinity for ECM acts as a size-matched control and, consistent with earlier data, shows that size alone can affect retention in the tumor (19). NJB2 and derivatives that have affinity for the tumor-specific domain of FN in the tumor microenvironment do show some additional extension of survival, although no mice survived and there was only a marginal increase in efficacy with increased affinity. We did not observe a large sink of nanobody–IL-2 in blood or spleen as has been observed for larger TA99-IL-2 fusions (5) presumably because the small size of nanobody–cytokine fusions leads to very rapid clearance from the circulation as reported previously (9).

### I.t. administration of anti-EIIIB immunocytokines enables high B16F10 cure rates

**Fig. 5.**
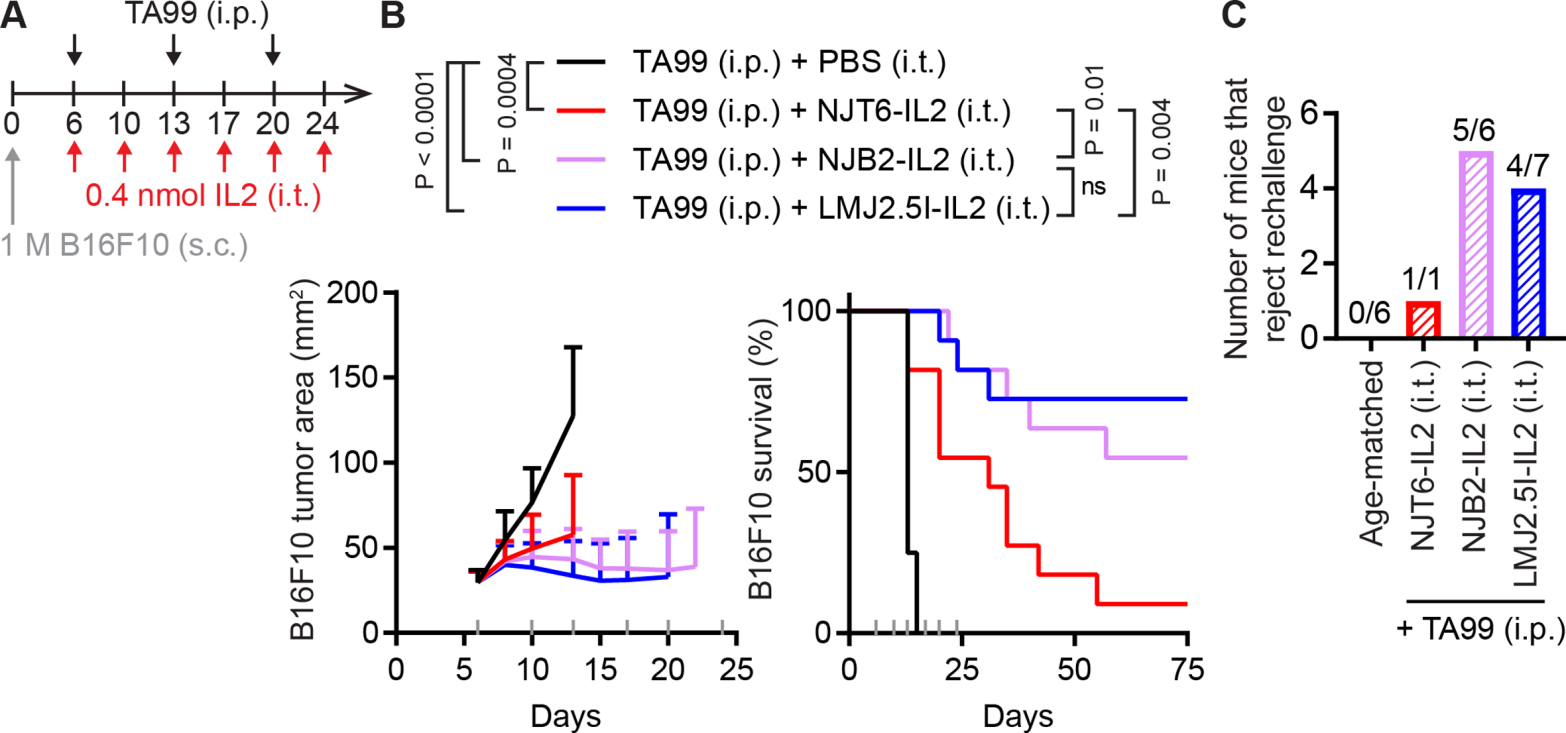
I.t. administration of EIIIB-specific IL-2 immunocytokines enables high B16F10 cure rate. (A) B16F10 study timeline. Mice were inoculated with 1 M B16F10 cells s.c. in the right flank on day 0. Mice were treated on indicated days with 100 μg TA99 (i.p.) and 0.4 nmol (12.8 μg) IL-2 fusions (i.t.). (B) Tumor growth (left) and survival (right). Gray ticks above the *x*-axis mark treatment days. Tumor area (mean + SD) is shown until a mouse in that group is euthanized. Survival comparisons were generated by a log-rank Mantel–Cox test. ns, not significant. *n* = 8 for TA99 + PBS, *n* = 11 for all other groups. (C) All surviving mice were rechallenged with 0.1 M B16F10 cells s.c. in the left flank on day 94 and tumor growth was monitored with no additional treatment. *n* = 6 for age-matched controls, *n* = 1 for TA99 + NJT6-IL2, *n* = 6 for TA99 + NJB2-IL2, *n* = 7 for TA99 + LMJ2.5I-IL2.

The intravenous administration of EIIIB-specific nanobody–IL-2 fusions resulted in only a mild nanobody-driven impact on survival and cellular biodistribution in two different solid tumor models (Figs 3 and 4). We next tested if avoiding systemic circulation altogether by switching from intravenous to i.t. administration could improve the impact of ECM affinity on IL-2 efficacy in the B16F10 model. Since i.t. administration inherently increases drug exposure at the tumor, we reduced the IL-2 dose from 1 to 0.4 nmol, and the frequency of IL-2 dosing from thrice weekly to twice weekly (Fig. 5A).

Even with a reduced IL-2 dose and frequency, i.t. administration of nanobody–IL-2 fusions to B16F10 tumors markedly reduced tumor growth and led to a more pronounced benefit from enhanced affinity for EIIIB (Fig. 5B). Compared to untargeted NJT6-IL2 (1/11 cures), we observed improved survival with nanomolar NJB2-IL2 (6/11 cures, *P* = 0.01) and picomolar LMJ2.5I-IL2 (8/11 cures, *P* = 0.004). Although LMJ2.5I-IL2 led to the highest cure rate, there was no statistical difference in survival between NJB2-IL2 and LMJ2.5I-IL2 (*P* = 0.49), indicating that picomolar and nanomolar targeting behave similarly in this setting. We did, however, observe enhanced tumor necrosis levels in mice treated with LMJ2.5I-IL2 (Figure S9) in line with the longer survival with this treatment. No weight loss was observed in treated mice (Figure S6C). When cured mice (surviving at 94 days) were rechallenged with 0.1 M B16F10 cells in the opposite flank, a majority of mice rejected rechallenge, indicating immunological memory from the combination of nanobody–IL-2 fusions and TA99 (Fig. 5C). Out of the 10 mice that rejected rechallenge, six developed vitiligo, which reflects an antimelanocyte response (20) (Figure S10). Further reducing the i.t. dose and frequency to 0.2 nmol once weekly failed to cure any tumors, indicating a dose-dependent response, but did lead to some extension of survival for the highest affinity immunocytokine (Figure S11). These data show that i.t. injection of small ECM-binding immunocytokines was more effective than systemic delivery.

## Discussion

Development of optimal criteria to design and dose cytokine constructs for cancer immunotherapy is an active area of study (18, 21–25). Proteins of the ECM, such as EIIIB+FN as described here, are promising targets because of their stability and their selective, abundant expression in disease sites. Although nanobody–cytokine fusions targeting cancer cell surface antigens have been studied previously in mice (26), here we develop and test ECM specific nanobody–cytokine fusions and characterize effective design parameters for such small-format immunocytokines. We engineered nanobody-IL-2 immunocytokines targeting the tumor ECM that are small in molecular size (∼32 kDa), possess a range of binding affinities to tumor-associated, EIIIB-containing FN (untargeted, nanomolar, and picomolar) and administered them via different routes (i.v. and i.t.) into immunocompetent mice.

The systemic delivery of IL-2 with picomolar affinity to EIIIB via i.v. injections resulted in a modest but statistically significant extension of survival compared to untargeted IL-2 in two different immunocompetent solid tumor models, B16F10 (combination with TA99) and 4T1 (monotherapy). However, LMJ2.5I-IL-2 extended median survival by only 2 days compared to untargeted IL-2, and all mice succumbed to tumor burden. In contrast, the i.t. administration of IL-2 immunocytokines with nanomolar or picomolar affinity to EIIIB resulted in strong extension of survival in mice bearing established B16F10 tumors (respectively 55% and 73% survival at 94 days, here designated as cures). High cure rate was not seen with i.t. NJT6-IL2, or with intravenous delivery of any of the nanobody-IL2 fusions, suggesting that both affinity to the tumor ECM and i.t. administration can together maximize antitumor benefit from small-format (∼32 kDa) IL-2 immunocytokines. Benefits of i.t. administration have also been observed previously for immunocytokines that bind tumor cellular targets (27).

We have shown previously that when using large IgG format immunocytokines based on TA99-IL-2 fusions (183 kDa), the IL-2 moiety and not the antibody specificity govern biodistribution and therapeutic efficacy (5). Since IL-2 engages with receptors on immune cells that are highly abundant in blood and lymphoid tissues, immunocytokine pharmacokinetics (PK) are dominated by the cytokine moiety after i.v. dosing. Indeed, the majority of large-format IL-2 immunocytokine is bound to IL-2-receptor-expressing cells in the systemic sink. PK modeling predicted that smaller immunocytokines might exhibit comparatively improved tumor-specificity and efficacy, due to their more rapid clearance from circulation and faster diffusion into the tumor microenvironment. In this work, we observed rapid clearance of the smaller (∼32 kDa) anti-EIIIB immunocytokines from circulation, and did not observe elevated binding to immune cells in the spleen. However, only a small percentage of immune cells in the tumor bound the small immunocytokines. Furthermore, IL-2 fusions with or without affinity to EIIIB had similar cellular biodistributions on immune cells. For small immunocytokines of the type studied here, sequestration by binding to IL-2 receptor on immune cells appears insufficient to explain their minimal efficacy, indicating that additional parameters still need to be considered in the context of systemic delivery. Previous tumor targeting theories have predicted that smaller agents with picomolar affinity could accumulate favorably within tumors (28). Further study of IL-2 immunocytokines of different sizes or dosing frequencies may reveal an optimum circulation time that allows sufficient tumor enrichment while maintaining minimal binding to the systemic sink. This optimum may or may not be feasibly approachable, depending on the systemic toxicity incurred by systemic dosing of such agents at high level.

Although in this work we only observed minor nanobody-driven benefits for small IL-2 immunocytokines delivered intravenously, there may be other situations where tumor-targeting could lead to larger improvements for systemically delivered cytokines. Others have reported efficacy and biodistribution improvements after i.v. dosing of anti-EIIIB-IL-2 immunocytokines (7, 29, 30), or nanobody-IL-2 fusions targeting other antigens like PD-L1 (26). B16F10 and 4T1 tumors express lower levels of EIIIB+FN compared to some other tumor models. Higher target expression may increase the degree of benefit conferred by tumor-targeting after intravenous dosing. The choice of cytokine may also impact these results, especially depending on cytokine affinity to its receptor and abundance of cytokine receptors in systemic circulation. Indeed, strategies aimed at minimizing systemic cytokine-mediated drug uptake include weakening the potency of the fused cytokine and converting the cytokine into a prodrug (31, 32). Several immunocytokines in clinical trials employ the former strategy by fusing a mutated IL-2 with reduced IL-2Rα binding to an antibody directed against tumor-associated antigens (33–36). Despite a weakened IL-2, systemic cytokine uptake appears still to be present, as indicated by high toxicity (33) and high uptake in nonpathological lymphoid tissues and spleens (34). Enabling effective systemic administration of immunocytokines is challenging as minimizing engagement in the periphery can often conflict with maximizing efficacy at the tumor. Further studies are needed with additional cytokines, antigens, and tumor models to clarify the degree of survival benefit conferred by attempted tumor-targeting of systemically administered immunocytokines. Testing immunocytokines in immunocompetent mice with fully size-matched untargeted controls will both be important aspects of studies intended to elucidate therapeutic index effects and systemic sink competition.

As an improved alternative to intravenous treatment, we dosed the nanobody-IL-2 fusions intratumorally. The i.t. setting enabled a high B16F10 cure rate and revealed larger differences between IL-2 with and without affinity to EIIIB. We and others (18, 37–39) find that beyond improving treatment efficacy, i.t. administration enables immunocytokine dose sparing and has been reported to reduce the formation of antidrug antibodies (27). Clinically, i.t. administration of wild-type IL-2—not as an immunocytokine—yielded impressive improvement in therapeutic efficacy against melanoma lesions and reduced toxicity (40, 41). Our current results demonstrate that immunocytokines are capable of unleashing antitumor effects superior to untargeted IL-2 when intratumorally injected.

Among the most studied ECM-specific IL-2-based immunocytokines is L19-IL2, consisting of a homodimerizing single-chain variable fragment directed against EIIIB-containing FN fused to wild-type IL-2. The administration of L19-IL2 and some other IL-2 immunocytokines (42, 43) in clinical trials has also shifted towards the i.t. route, likely to benefit from the improved efficacy that i.t. administration enables. I.t. administration is feasible for many histological conditions and target organs in the clinic (44), and effective design criteria for i.t. therapy are being increasingly understood (19). To further improve clinical translation, it will also be useful to study how to reduce i.t. dosing frequency, which may be accomplished by higher doses or increasing the size of the immunocytokine (19).

One theoretical advantage of systemic over i.t. delivery is the potential to deliver immunocytokines to distant metastases, since even small metastases also exhibit tumor-specific ECM changes such as EIIIB+FN (9). We have not tested that possibility in the experiments described here but prior studies have suggested that i.t. delivery can elicit abscopal effects on distant sites (18, 38) via priming of NK or T cell responses. Further research into both i.t. and systemic routes of delivery will be necessary to explore strategies to elicit both local effects at the primary site and efficacy against distant metastases.

ECM-targeting has many applications beyond cytokine therapy. The parent nanobody NJB2 has been shown to be a powerful tool for noninvasive in vivo imaging in several tumor models (9) and NJB2-based CAR T-cells were effective against B16F10 solid tumors (10). With an improved picomolar K_D_, the novel higher affinity nanobodies such as LMJ2.5I may enable tumor imaging over longer time-periods with high signal-to-noise resolution (45), or improved CAR T-cell efficacy. NJB2 and the higher affinity nanobodies such as LMJ2.5I can also be applied to the targeted delivery of small molecule drugs and radiopharmaceuticals where rapid systemic clearance enabled by small size and long on-target retention enabled by a slow off-rate are favorable (46). Future studies are needed to test these nanobodies in these contexts.

In this work, we generated ECM-specific immunocytokines based on IL-2 coupled to EIIIB-specific nanobodies. For these small (∼32 kDa) IL-2 immunocytokines, affinity to EIIIB provided only small advantage when intravenously injected in the models tested. In contrast, we show that the binding of these immunocytokines to the tumor ECM following i.t. delivery resulted in a durable antitumor immune response and resistance to subsequent tumor challenge. Future studies to extrapolate this finding to small immunocytokines directed against different tumor-associated targets and fused to other cytokines are still required. Nonetheless, we identify that i.t. administration can overcome the pharmacokinetic challenges of i.v. injected immunocytokines and drive major enhancements in survival.

## Materials and Methods

### Experimental design

The primary objective of this work was to evaluate how the therapeutic efficacy of small-format IL-2 immunocytokines is affected by target binding affinity and administration route. In order to control for therapeutic impacts from molecular weight, all nanobody-IL-2 fusions were size-matched (ranging from 31.8 to 32.2 kDa). In order to account for the impact from systemic IL-2-receptor-expressing cells, we only used immunocompetent mice. Tumor-bearing mice were randomized to ensure all groups had equal average tumor size at the start of treatment. All experiments were performed with at least five mice per group, and up to 11 mice per group, with the number of mice in each experiment stated in the legend. Our primary readout of therapeutic efficacy was survival. End-point criteria for euthanasia (tumor area exceeding 100 mm^2^) was predetermined before study initiation.

### Mice

Syngeneic mice for B16F10 experiments were purchased from Taconic (C57BL/6NTac) and The Jackson Laboratory (C57BL/6 J). Balb/C mice for 4T1 experiments were purchased from Taconic (BALB/cAnNTac). All animal work was conducted under the approval of the Massachusetts Institute of Technology Committee on Animal Care in accordance with federal, state, and local guidelines.

### Cells

B16F10 and 4T1 cells were purchased from ATCC and cultured according to vendor instructions. HEK293-F cells were purchased from Life Technologies and cultured in FreeStyle293 Expression Medium (Life Technologies). CTLL-2 cells were purchased from ATCC and cultured in RPMI-1640 (ATCC) supplemented with 10% fetal bovine serum (Gibco), 10% T cell culture supplement with concanavalin A (T-STIM with ConA, Corning), 20 mM HEPES, 1 mM sodium pyruvate, 0.05 mM 𝛽-mercaptoethanol (Life Technologies), 100 units/mL penicillin (Life Technologies), 100 µg/mL streptomycin (Life Technologies), 2 mM L-alanyl-L-glutamine (Life Technologies), and 1x minimal essential medium nonessential amino acids (Corning). All cells in culture were maintained at 37°C and 5% CO_2_. All cell lines tested negative for mycoplasma.

### In vivo tumor survival

Female mice, 7∼8-week-old, were inoculated on day 0 with B16F10 tumors (1 M cells in 50 μL PBS injected subcutaneously in the right flank) or 4T1 tumors (0.5 M cells in 50 μL PBS injected in the mammary fat pad). Treatments initiated on day 6 when tumors were established (average 25 mm^2^ for B16F10 and 15 mm^2^ for 4T1). TA99 was dosed i.p. at 100 μg in 100 μL PBS once per week. For intravenous IL-2 treatments, 1 nmol (32 μg) of nanobody-IL-2 fusion in 70 μL PBS was injected retro-orbitally thrice per week. For i.t. IL-2 treatments, 0.4 nmol of nanobody-IL-2 fusion in 20 μL PBS was injected intratumorally twice per week. Mice underwent 2 to 3 weeks of treatment, as outlined in each experimental figure. Tumor area (length × width) and body weight were recorded three times per week. Mice were euthanized when their tumor area exceeded 100 mm^2^. Cured mice (surviving at 94 days) along with age-matched control mice were rechallenged on day 94 with 0.1 M B16F10 cells in 50 μL PBS injected subcutaneously (s.c.) in the left flank and tumor growth was monitored.

### Cellular biodistribution

Similar to Tzeng et al (5), 7-week-old female mice were inoculated on day 0 with 1 M B16F10 cells in 50 μL PBS injected s.c. in the right flank. On day 8, mice were treated retro-orbitally with 1 nmol AF647-labeled nanobody-IL-2 in 70 µL PBS. A total of 24 hours later, blood was collected via cheek bleed into K2 EDTA tubes (Greiner Bio-one 450480). Mice were euthanized and tumors, tdLNs, and spleens were harvested and weighed. Tumors and tdLNs were mechanically dissociated, then incubated in RPMI 1 mg/mL Collagenase/Dispase (Sigma 11097113001), 20 μg/mL DNase I (Sigma 10104159001). Organs were rendered into single-cell suspension by filtration through 70-µm mesh screens. Spleens and blood were resuspended in ACK Lysing Buffer (Gibco A1049201). Cells were stained with Zombie Aqua viability dye (BioLegend 423101), then blocked with CD16/CD32 antibody (eBioscience Clone 93). In one panel, cells were stained with the antibodies APC/Cy7-CD45 (BioLegend 30-F11), PE/Cy7-CD3 (BioLegend 17A2), BV421-CD19 (BioLegend 6D5), FITC-NK1.1 (BioLegend PK136), BV605-CD8a (BioLegend 53–6.7), BUV737-CD4 (BD Biosciences GK1.5), and PE-FOXP3 (BioLegend 150D). In a separate panel, cells were stained with the antibodies APC/Cy7-CD45 (BioLegend 30-F11), PE/Cy7-Ly6C (BioLegend HK1.4), BV421-CD11b (BioLegend M1/70), BUV737-I-A/I-E (BD Biosciences M5/114), FITC-F4/80 (BioLegend BM8), BV605-CD24 (BioLegend M1/69), and a PE-dump channel with PE-CD3, PE-NK1.1, PE-CD19, and PE-FOXP3 (BioLegend, same clones as above) (47). Cells were fixed and intracellular staining was performed in Permeabilization Buffer (Invitrogen) according to manufacturer’s instructions. Samples were run on a BD LSRFortessa HTS-1 analyzer and data were analyzed with FlowJo software (V10.4). Gating strategy is shown in Figure S7. When reporting median AF647, background levels from PBS mice were subtracted.

### Hematoxylin and eosin staining

Mice were inoculated on day 0 with 1 M B16F10 cells in 50 μL PBS injected s.c. in the right flank. Mice were treated with 100 μg TA99 (i.p.) on day 6, and PBS or 0.4 nmol nanobody-IL2 fusions (i.t.) on days 6 and 10. On day 12, tumors were excised and fixed in 4% formaldehyde in PBS at RT overnight and paraffin-embedded following standard procedures. Consecutive sections (4 to 6 μm) were prepared using a Leica RM2255 rotary microtome, dried at 60°C for 1 hour. Hematoxylin and eosin (H&E) staining was performed using standard protocols with the help of a Thermo Scientific Shandon Varistain Gemini ES Automated Slide Stainer. A Leica Apeiro AT2 slide scanner was used for image documentation.

### Yeast surface display

The antigen for yeast display was the protein fragment of FN splice variant EIIIB, previously made in the Hynes laboratory (48, 49). EIIIB was biotinylated using ChromaLink NHS-biotin reagent (Solulink) following manufacturer’s protocol. Yeast selections were performed following previous protocols (14, 50, 51). An initial yeast library (diversity 4 × 10^7^) was generated via 20∼40 cycles of error-prone PCR (using 2 μM each of 8-oxo-dGTP and dPTP) on plasmid DNA containing NJB2. The nanobody library was displayed on the surface of *Saccharomyces cerevisiae* (yeast) strain EBY100 using the pCTcon2 plasmid, resulting in expression of Aga2p–HA tag–(G_4_S)_3_ linker–nanobody–G_3_S linker–cMyc tag. The yeast library went through three initial rounds of equilibrium sorting (sorts 1.2, 1.3, and 1.4), where yeast were incubated in PBS 0.1% BSA with 10 nM biotinylated EIIIB, chicken-anti-cMyc (Exalpha) and mouse-anti-HA (BioLegend 16B12) for at least 1 hour. Yeast were washed, and secondarily stained with Streptavidin Alexa Fluor 647 (Invitrogen S21374), goat-antichicken Alexa Fluor488 (Invitrogen A11039), and goat-antimouse PE (Invitrogen P-852) for 30 minutes. Yeast were washed and sorted on a BD FACSAria III Cell Sorter. The top 0.1∼1% of yeast were sorted for binding to EIIIB, as determined by Streptavidin-AF647 (Figure S2B). Sorted yeast were grown up for the next round of sorts. After three initial equilibrium sorts, a second yeast library (diversity 6 × 10^7^) was generated as before, but now with 60 cycles of error-prone PCR on an equimolar mixture of plasmid DNA containing clones LMJ1.2C, LMJ1.2 G, and LMJ1.3 J. The second library went through equilibrium sorts as before, but at 50 nM and 0.5 nM biotinylated EIIIB (sorts 2.2 and 2.3, respectively). We next performed kinetic sorts, where yeast were incubated in PBS 0.1% BSA with 50∼100 nM biotinylated EIIIB for at least 1 hour, washed, then resuspended in 100 nM unlabeled EIIIB at 4°C for 24 hours (sort 2.4), 4°C for 72 hours (sort 2.5), or room temperature for 72 hours (sort 2.6). In sorts 2.5 and 2.6, we also gated for equal expression of HA and cMyc to prevent selective pressure for mutations in cMyc. After each sort, plasmid DNA of sorted yeast was isolated using Zymoprep Yeast Plasmid Miniprep II Kit (Zymo research). Isolated plasmid DNA was transformed into Stellar Competent cells to isolate individual colonies for Sanger sequencing. To visualize mutations, the structure of the NJB2 nanobody was predicted using ABodyBuilder on the SAbPred server (15, 16) based on PDB 7KKJ (52). Graphics were generated using UCSF Chimera (17).

### Nanobody subcloning, expression, and purification in WK6 cells

The engineered nanobody sequences were subcloned from the yeast display vector pCTcon2 into the pHEN6 periplasmic expression vector with a C-terminal LPETG sortase motif followed by a 6-His tag. Sequences are shown in Table S1. Proteins were expressed in WK6 *Escherichia coli* cells. A total of 1 mM IPTG was used to induce protein expression at OD600 = 0.6 (16 hours at 30°C). The His-tagged nanobody present in the periplasmic fraction was extracted by osmotic shock and purified using Ni-NTA beads (Qiagen). To confirm the molecular weights, proteins were run alongside the Precision Plus Kaleidoscope Prestained Protein standards (Biorad) on a Novex 4% to 20% Tris-Glycine gel and stained in Coomassie blue stain. The purified protein was buffer-exchanged into PBS and concentrated using 10 K Amicon filters (EMD Millipore), flash frozen in liquid nitrogen and stored at −80°C. Nanobodies expressed in WK6 cells were only used in in vitro assays for EIIIB binding affinity and specificity.

### Affinity determination by BLI

To determine the affinities of the recombinant nanobodies, BLI was done using a ForteBio Octet RED96 biolayer interferometer (Pall ForteBio). Streptavidin-coated BLI biosensor tips (ForteBio) were soaked in the assay running buffer [PBS with 0.05% Tween-20 and 1% recombinant human albumin (Sigma)] for 10 minutes. Biotinylated EIIIB was then immobilized on streptavidin-coated BLI biosensor tips by immersion in a 2 μg/mL solution. The association and dissociation were analyzed for different concentrations of analyte ranging from 0.1 to 350 nM. Association and dissociation rate constants were determined using the ForteBio data analysis software (V8.2) using the 1:1 binding model and a global fit analysis with double referencing.

### C-terminal sortase tagging

C-terminal sortase tagging with biotin was done using the *Staphylococcus aureus* sortase A (pentamutant variant 5M-SrtA). The sortase was expressed and purified as previously described (53). For biotin tagging, sortase (5 μM) was incubated with purified His-tagged nanobodies (150 μM) and nucleophile (GGGK-Biotin, 500 μM) in sortase buffer containing 50 mM Tris·HCl, pH 7.5, 150 mM NaCl, 10 mM CaCl_2_ for 3 to 12 hours at 4°C. The unreacted sortase and His-tagged nanobody were removed by incubation with Ni-NTA beads with agitation for 5 minutes at 25°C followed by centrifugation. The biotin-tagged nanobodies were buffer-exchanged into PBS and concentrated using 3 K 0.5 mL Amicon filters (EMD Millipore) and stored at −20°C with 5% glycerol.

### Immunoblotting

Samples including recombinant proteins, murine plasma FN (Abcam) and human plasma FNs (BD Biosciences), and in-house ECM-enriched samples from murine lung were prepared in Laemmli buffer containing 100 mM dithiothreitol. All proteins were separated by SDS-PAGE on 4% to 20% Tris-Glycine gradient Gels (Novex) and transferred onto nitrocellulose membranes (Millipore, Billerica). Immunoblotting was performed using biotin-tagged nanobodies. Following primary antibody incubation, the membranes were washed and incubated in the presence of HRP-Streptavidin (BD Biosciences). Finally, membranes were washed and incubated with Western Lightning Chemiluminescence Reagent (PerkinElmer LAS).

### ELISA

96-well ELISA plates (Thermo Fisher Scientific) were coated with 3 μg/mL of EIIIB-His protein overnight at 4°C. The coated wells were washed with PBST (PBS with 0.05% Tween-20) and blocked with blocking solution containing PBST and 5% nonfat dry milk (Biorad). Dilutions of biotinylated nanobodies were made in blocking solution, added to the coated wells and incubated for 1 hour at RT. Following washes with PBST, wells were incubated with Streptavidin-HRP (BD Pharmingen) at a 1:1,000 dilution for 1 hour, washed and incubated with chromogenic substrate TMB (Sigma) until blue color developed. The reaction was stopped with 1 N HCl and absorbance at 450 nm was measured using the Infinite M200 PRO microplate reader (Tecan).

### Cloning and protein production of therapeutic proteins

The nanobody cDNA was amplified by PCR and fused with murine IL-2 cDNA containing the C-terminal LPETG sortase motif followed by a 6-His tag. The fusions were cloned by In-Fusion snap assembly kits (Takara Bio) into the gWIZ vector (Genlantis). Sequences are shown in Table S2. Plasmids were transformed and amplified in Stellar competent cells (generated in-house) and purified using NucleoBond Xtra endotoxin-free Midi prep kit (Macherey–Nagel). A 0.22-μm sterile filtered plasmid DNA encoding each protein was transfected into suspension HEK293 cells with Polyethylenimine (Polysciences 23966) in OptiPRO Serum Free Medium (Thermo Fisher). Nanobody–IL-2 fusions were purified using TALON Metal Affinity Resin (Takara Bio). TA99 was purified using rProtein A Sepharose Fast Flow Resin (Cytiva). Some IL-2 fusions were further purified by size-exclusion chromatography using a Superdex 200 Increase 10/300 GL column on an ÄKTA FPLC (GE Healthcare). All proteins were buffer-exchanged into sterile PBS (Corning), 0.22-μm sterile filtered (Corning), and confirmed for minimal endotoxin (< 0.1 EU per dose) as measured by a LAL Chromogenic Endotoxin Quantitation Kit (Pierce). To confirm molecular weight, proteins were run alongside a Novex Sharp Pre-Stained Protein Standard on an NuPAGE 4% to 12% Bis-Tris gel (Invitrogen) in MES running buffer and stained in SimplyBlue SafeStain (Life Technologies). Proteins were flash frozen in liquid nitrogen and stored at −80°C.

### CTLL-2 proliferation assay

CTLL-2 cells were seeded onto 96-well tissue culture plates at 5,000 cells/well in 100 μL of media without T-STIM and without ConA. Cells were cultured for 48 hours with varying concentrations of nanobody-IL-2 fusions. Cell proliferation was determined by WST-1-based colorimetric assay (Roche) according to manufacturer’s instructions. Absorbance at 450 nm with reference absorbance at 650 nm was measured using an Infinite M1000 microplate reader (Tecan).

### Fluorescently-labeled nanobody–IL-2 fusions

Fluorescently labeled proteins were prepared by incubating proteins (1 mg/mL in PBS with 0.1 M K_2_HPO_4_, pH 9) with 6 fold molar excess of AF647 NHS ester (Invitrogen A20006) for 1.5 hours at room temperature in the dark. Free dye was removed using 10 K Amicon filters (EMD Millipore) and two successive PD SpinTrap G-25 columns (Cytiva). Dye to protein ratios ranged from 1.5 to 2. Fluorescently labeled and unlabeled nanobody–IL-2 fusions were prepared such that each dose contained 1 nmol nanobody–IL-2 and 1.5 nmol dye.

### Statistical analysis

Statistical analysis was performed with GraphPad Prism software (V7). Survival curves were compared by log-rank Mantel–Cox test. As described in figure legends, comparisons between groups were assessed by one-way or two-way analysis of variance (ANOVA) with Tukey’s multiple comparisons test. The *n*-values are indicated in figure legends and *P-*values are shown in the figures.

## Supplementary Material

pgac244_Supplemental_File

## Data Availability

The data that support the findings of this study are available in this manuscript and the Supplementary Material.

## Notes

Classification: Biological, Health, and Medical Sciences; Applied Biological Sciences.

**Competing Interest:** R.O.H., K.D.W., N.J., N.M., and E.A.L. are named as inventors in a patent application filed by the Massachusetts Institute of Technology related to the data presented in this work (Application number 63/304954). R.O.H. and N.J. are named as inventors in a patent application filed by the Massachusetts Institute of Technology related to the data presented in this work (US20190225693A1). The remaining authors report no competing interests.
