# Supplementary material for: Intratumoral nanobody–IL-2 fusions that bind the tumor extracellular matrix suppress solid tumor growth in mice
Source: PNAS Nexus. 2022 Nov 3;1(5):pgac244. doi: 10.1093/pnasnexus/pgac244 (PMC9802395; doi:10.1093/pnasnexus/pgac244)
Supplement: pgac244_Supplemental_File [file pgac244_supplemental_file.pdf]

PNAS nexus

Supplementary Information for
Intratumoral nanobody-IL-2 fusions that bind the tumor extracellular matrix suppress solid tumor growth in mice

Emi A. Lutz,† Noor Jailkhani,† Noor Momin,† Ying Huang, Allison Sheen, Byong H. Kang, K. Dane Wittrup,* Richard O. Hynes*

†Co-first authors: Emi A. Lutz; Noor Jailkhani; Noor Momin

*Co-corresponding authors: K. Dane Wittrup; Richard O. Hynes

Email: wittrup@mit.edu; rohynes@mit.edu

This PDF file includes:

Figures S1 to S11
Tables S1 to S2

A

```
IL2-human-wt  APTSSS--------------TKKTQLQLEHLLLDLQMILNGINNYKNPKLTRMLTFKFYM  46
IL2-mouse-wt  APTSSSTSSSTAEAQQQQQQQQQQQQHLEQLLMDLQELLSRMENYRNLKLPRMLTFKFYL  60
IL2-mouse-mt  APTSSSTSSSTAEAQQQQQQQQQQQQHLEQLLMDLQELLSRMENYRNLKLPDMLTFEFYL  60

IL2-human-wt  PKKATELKHLQCLEEELKPLEEVLNLAQSKNFHLRP-RDLISNINVIVLELKGSETTFMC 105
IL2-mouse-wt  PKQATELKDLQCLEDELGPLRHVLDLTQSKSFQLEDAENFISNIRVTVVKLKGSDNTFEC 120
IL2-mouse-mt  PKQATELKDLQCLERELGPLRHVLDLTQSKSFQLEDAENFISNIRRTVVKLKGSDNTFEC 120

IL2-human-wt  EYADETATIVEFLNRWITFCQSIISTLT- 133
IL2-mouse-wt  QFDDESATVVDFLRRWIAFCQSIISTSPQ 149
IL2-mouse-mt  QFDDESATVVDFLRRWIAFCTSIISTSPQ 149
```

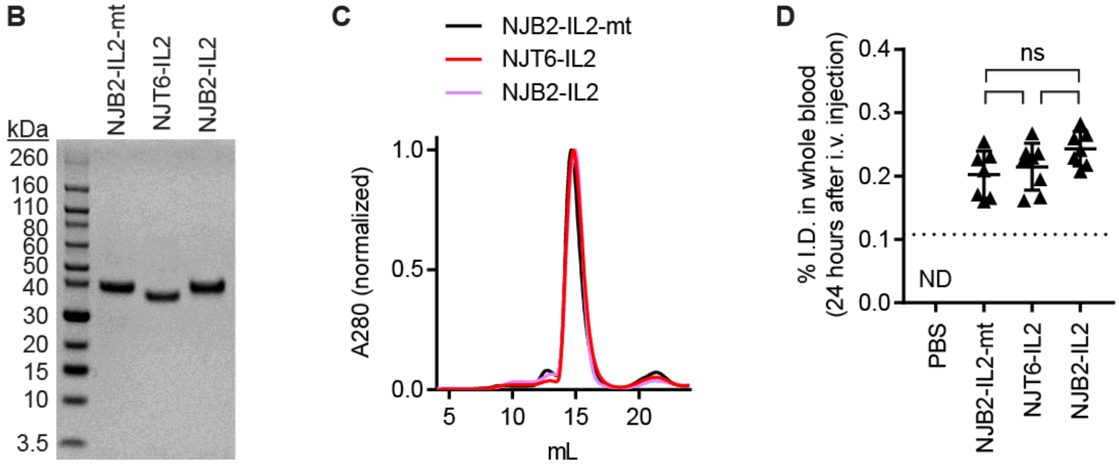

Fig. S1. Characterization of NJB2-IL2-mt, NJT6-IL2, and NJB2-IL2. (**A**), An alignment of wild-type human IL-2, wild-type murine IL-2, and inactive murine IL2-mt. The five mutations used to generate IL2-mt are highlighted. The three mutations highlighted in blue are described by Vazquez-Lombardi et al (*11*) to disrupt binding to the alpha subunit of human IL-2. The two mutations in red are described by Liu et al (*12*) to disrupt binding to the beta and gamma subunits of human IL-2. (**B**), NJB2-IL2-mt (31.8 kDa), NJT6-IL2 (32.2 kDa), and NJB2-IL2 (31.8 kDa) on a non-reducing SDS-PAGE gel with Coomassie blue stain. (**C**), Size-exclusion chromatography of indicated proteins using a Superdex200 Increase 10/300 GL column in PBS. (**D**), Mice were injected i.v. with PBS or Alexa Fluor 647-labeled immunocytokines, as described in Fig. 4. 24 hours later, blood was collected via cheek bleed and fluorescence of whole blood was measured. % injected dose (I.D.) was determined based on a standard curve of fluorescently-labeled immunocytokines added to blood from untreated mice *ex vivo*, and the assumption that the blood compartment in a mouse is 2 mL. Dotted line shows the limit of detection. ND, no fluorescence detected; ns, not significant. Mean ± SD; n = 7~8. Data were analyzed with one-way analysis of variance (ANOVA) with Tukey's multiple comparisons test.

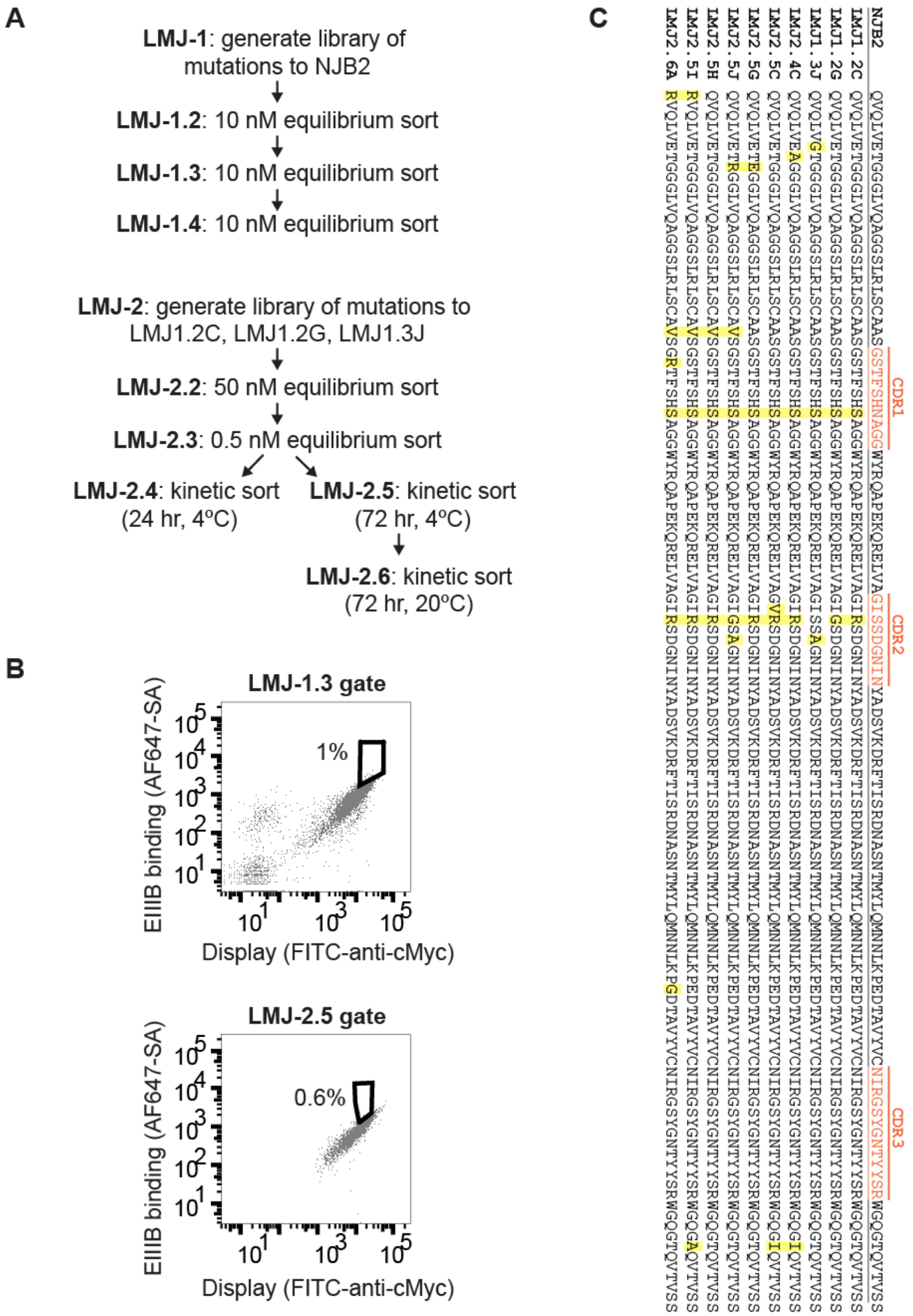

Fig. S2. Yeast surface display strategy and clones. (**A**), Pipeline for yeast surface display is outlined. (**B**), Representative sorting gates (black) are shown for yeast display libraries (gray). We stringently collected the top 0.1~1% of the library during each sort. (**C**), Sequences are shown for nanobody clones that were generated by yeast surface display and chosen for recombinant expression. Nanobodies were chosen if they contained mutations that were observed in many clones after yeast display sorts. Highlighted residues indicate mutations compared to the parental NJB2 nanobody.

A

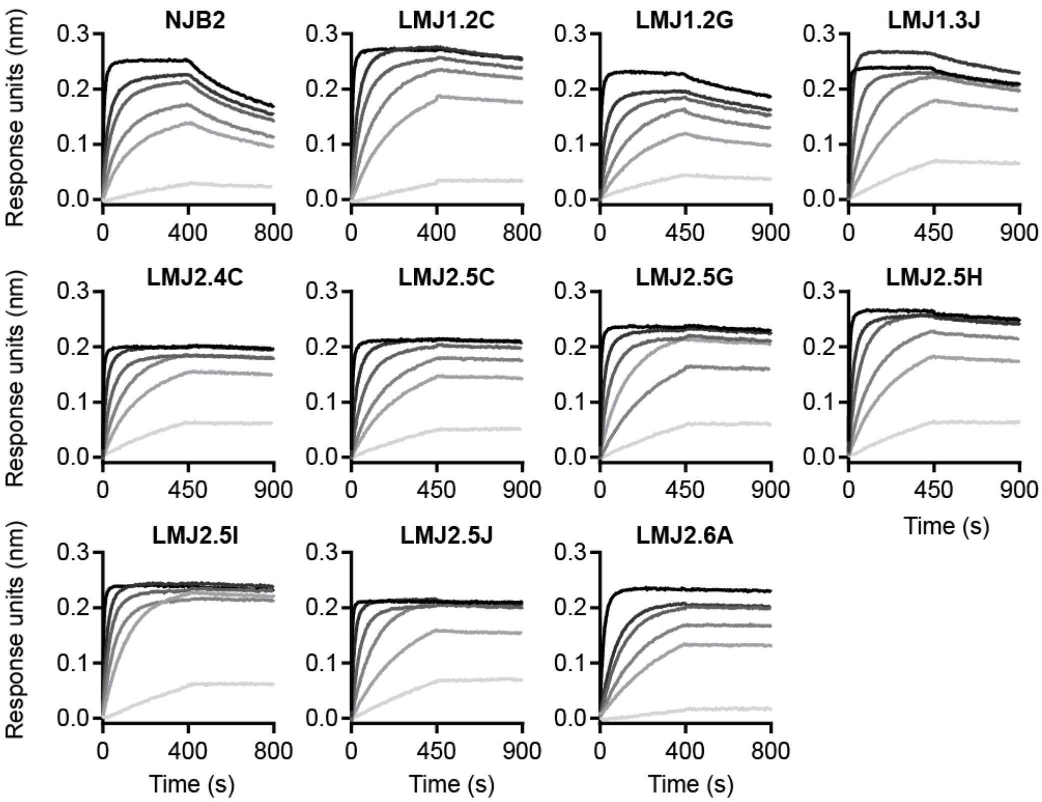

B

Protein	K_D (M)	k_{on} (M^{-1} s^{-1})	k_{off} (s^{-1})	χ^2
NJB2	1.82 E-09	5.69 E+05	1.04 E-03	0.118
LMJ1.2C	3.00 E-10	4.99 E+05	1.50 E-04	0.0845
LMJ1.2G	9.73 E-10	4.88 E+05	4.75 E-04	0.1045
LMJ1.3J	4.02 E-10	7.58 E+05	3.05 E-04	0.0291
LMJ2.4C	6.26 E-11	7.68 E+05	4.80 E-05	0.1123
LMJ2.5C	7.85 E-11	4.49 E+05	3.52 E-05	0.1174
LMJ2.5G	1.66 E-10	5.52 E+05	9.13 E-05	0.5913
LMJ2.5H	2.27 E-10	5.98 E+05	1.36 E-04	0.164
LMJ2.5I	2.54 E-11	9.82 E+05	2.49 E-05	0.1555
LMJ2.5J	6.22 E-11	7.16 E+05	4.45 E-05	0.0293
LMJ2.6A	3.44 E-10	2.17 E+05	7.47 E-05	0.1215
NJB2-IL2	9.96 E-10	2.90 E+05	2.89 E-04	0.1875
LMJ1.2C-IL2	1.44 E-11	3.50 E+05	5.06 E-06	0.5584
LMJ2.5I-IL2	<1.0 E-12	2.20 E+05	<1.0 E-07	0.2104

Fig. S3. Bio-layer interferometry of nanobody clones. (**A**), Biotin-tagged nanobodies were analyzed by bio-layer interferometry (BLI) using streptavidin tips coated with biotinylated EIIIB. Dark to light indicates analyte concentration of 350, 80, 30, 10, 4, and 1 nM for LMJ1.2G, LMJ1.3J, LMJ2.4C, LMJ2.5C, LMJ2.5G, LMJ2.5H, and LMJ2.5J. Dark to light indicates analyte concentrations of 350, 70, 35, 14, 7, and 0.7 nM for NJB2, LMJ1.2C, LMJ2.5I, and LMJ2.6A. (**B**), Equilibrium dissociation constant (K_D), rate of association (k_{on}), rate of dissociation (k_{off}), and X^2 values from BLI data are reported using 1:1 curve fits.

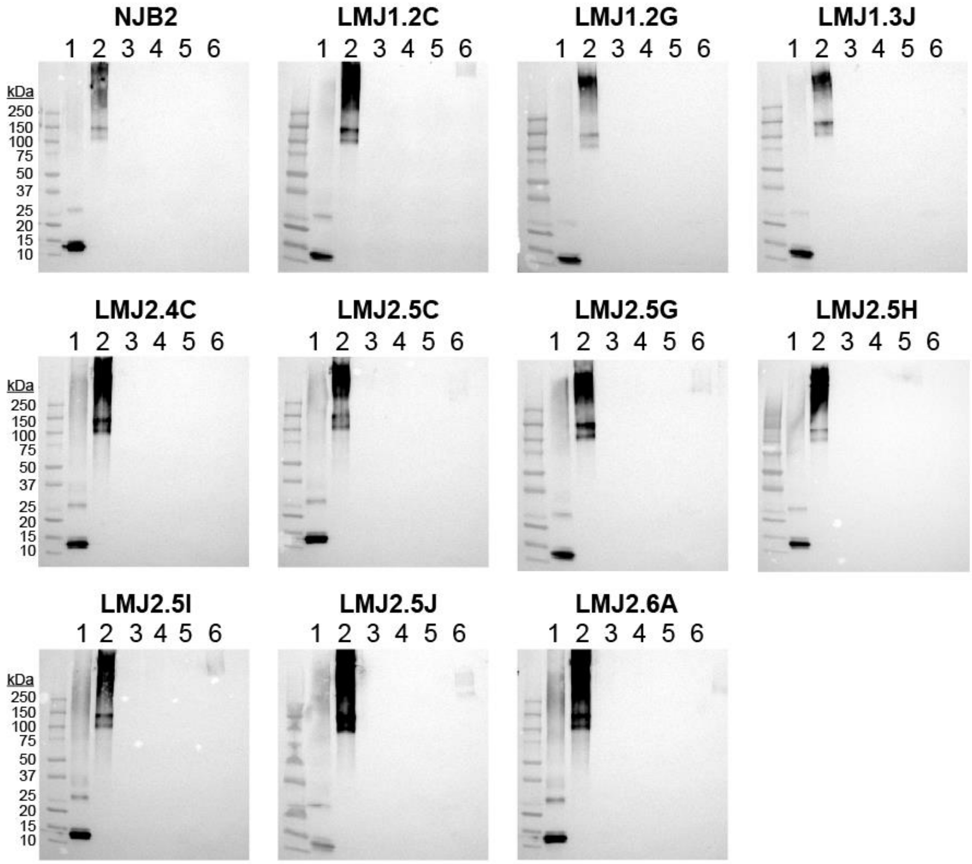

Fig. S4. Immunoblot of nanobody clones. The specificities of the biotin-tagged nanobodies were analyzed by immunoblot. Lanes 1-6, (1) EIIIB-His6, (2) FN 7-15 EIIIB (3) human plasma FN (4) mouse plasma FN (5) normal murine lung ECM (6) His-GFP protein.

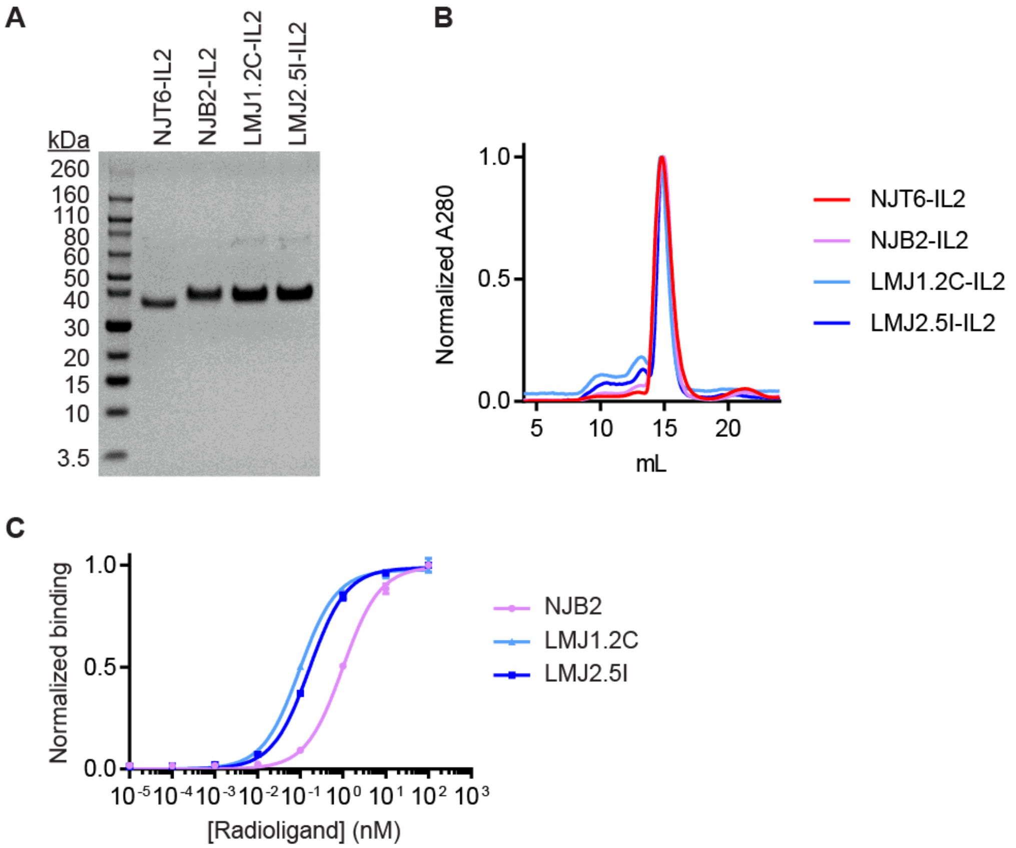

Fig. S5. Characterization of NJT6-IL2, NJB2-IL2, LMJ1.2C-IL2, and LMJ2.5I-IL2. (**A**), NJT6-IL2 (32.2 kDa), NJB2-IL2 (31.8 kDa), LMJ1.2C-IL2 (31.9 kDa), and LMJ2.5I-IL2 (31.9 kDa) on a non-reducing SDS-PAGE gel with Coomassie blue stain. (**B**), Size-exclusion chromatography of indicated proteins using a Superdex200 Increase 10/300 GL column in PBS. (**C**), Biotin-tagged nanobodies were analyzed by ELISA on EIIIB-coated plates (mean ± SD; *n* = 3).

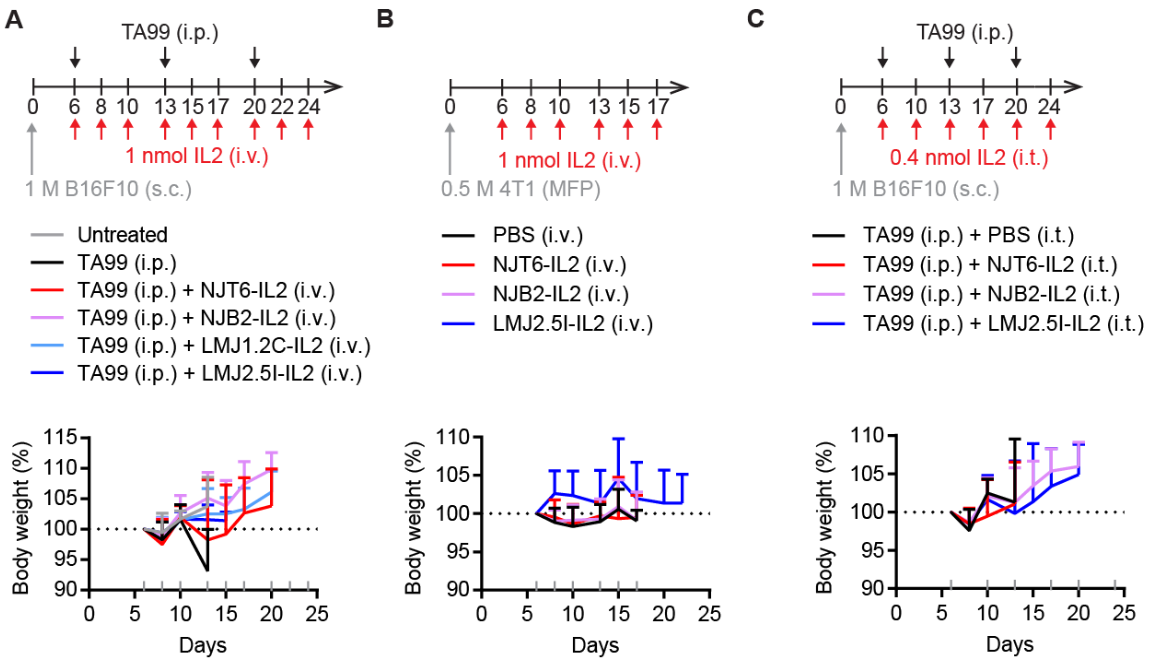

Fig. S6. No IL-2 related weight loss is observed in mice. Study timeline (top) and mouse body weight (bottom). Body weight as a percentage of day 6 body weight (mean + SD) is shown until a mouse in that group is euthanized. Gray ticks above the x-axis mark treatment days. (**A**), B16F10 intravenous study (associated with Fig. 3, A and B). *n* = 7~9. (**B**), 4T1 intravenous study (associated with Fig. 3, C and D). *n* = 6~7. (**C**), B16F10 intratumoral study (associated with Fig. 5, A and B). *n* = 8 for TA99 + PBS, *n* = 11 for all other groups.

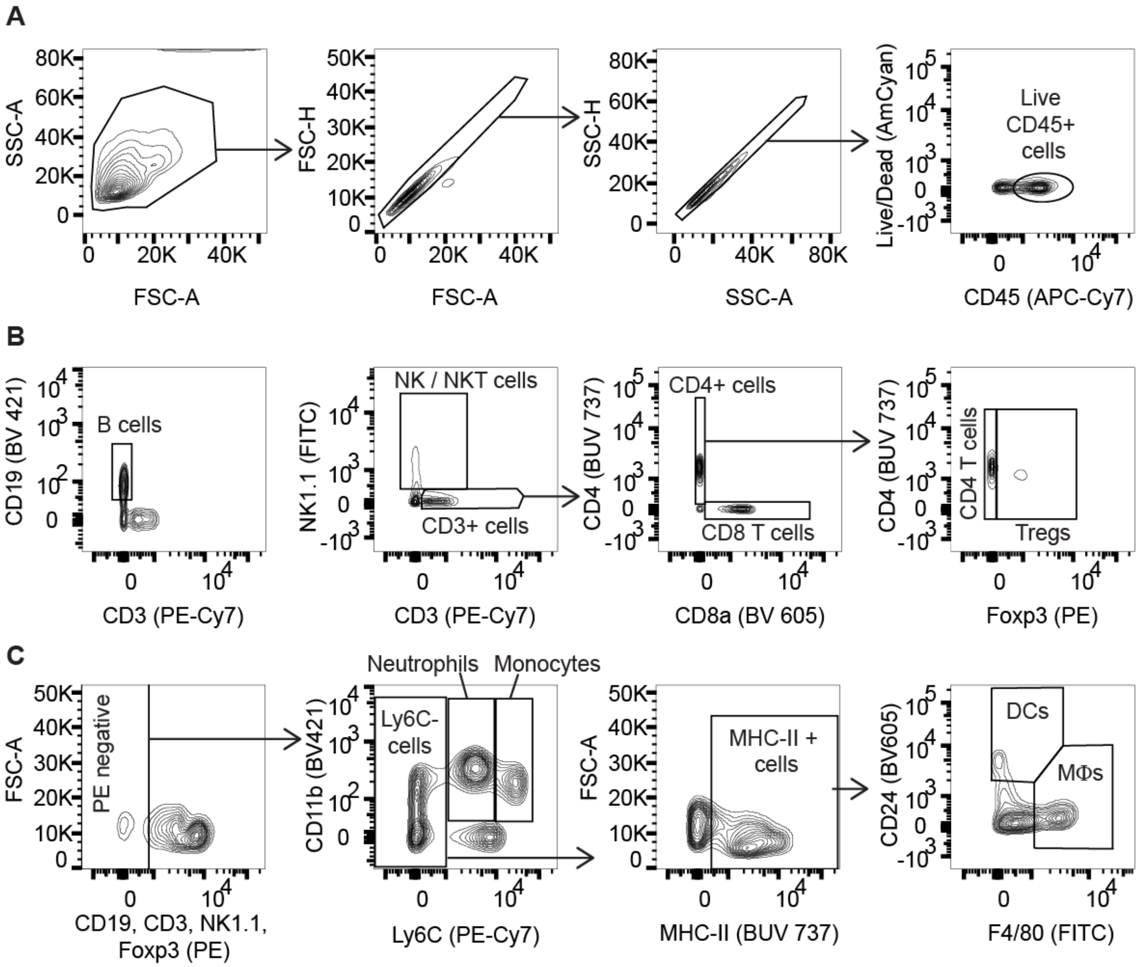

Fig. S7. Flow cytometry gating for cellular biodistribution. (**A**), To process flow cytometry data, cells were first gated for single cells, then gated for live CD45+ cells. (**B**), In one flow panel, live CD45+ cells were further gated into NK/NKT cells and T cell subsets. (**C**), In a separate flow panel, live CD45+ cells were gated for neutrophils, monocytes, dendritic cells (DCs), and macrophages (MΦs). The first gate excluded cells that expressed markers already used in the first panel.

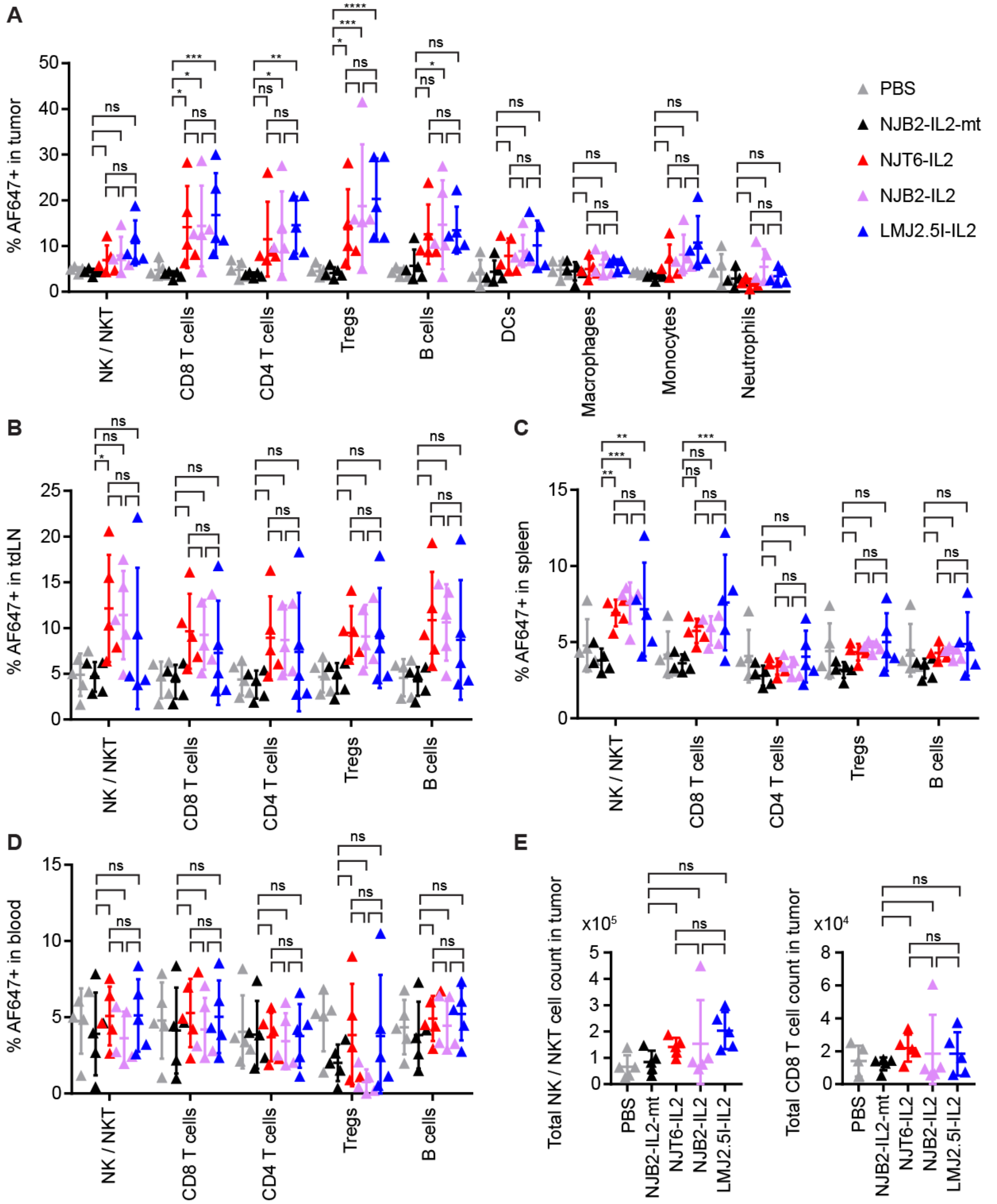

Fig. S8. Cellular biodistribution and cell counts after intravenous treatment. Mice were inoculated with 1 M B16F10 cells subcutaneously in the right flank on day 0. Mice were treated on day 8 with 1 nmol (32 μg) AF647-labeled IL-2 fusions (i.v.). 24 hours later, mice were sacrificed for necropsy and flow cytometry. % $AF647^{+}$ in different cell populations are shown in the (**A**), tumor, (**B**), tumor draining lymph node, (**C**), spleen, and (**D**), blood. (**E**), Total cell count in the tumor for NK/NKT cells (left) and $CD8^{+}$ T cells (right). All data are shown as mean ± SD; n = 5. Data were analyzed with two-way analysis of variance (ANOVA) with Tukey's multiple comparisons test.

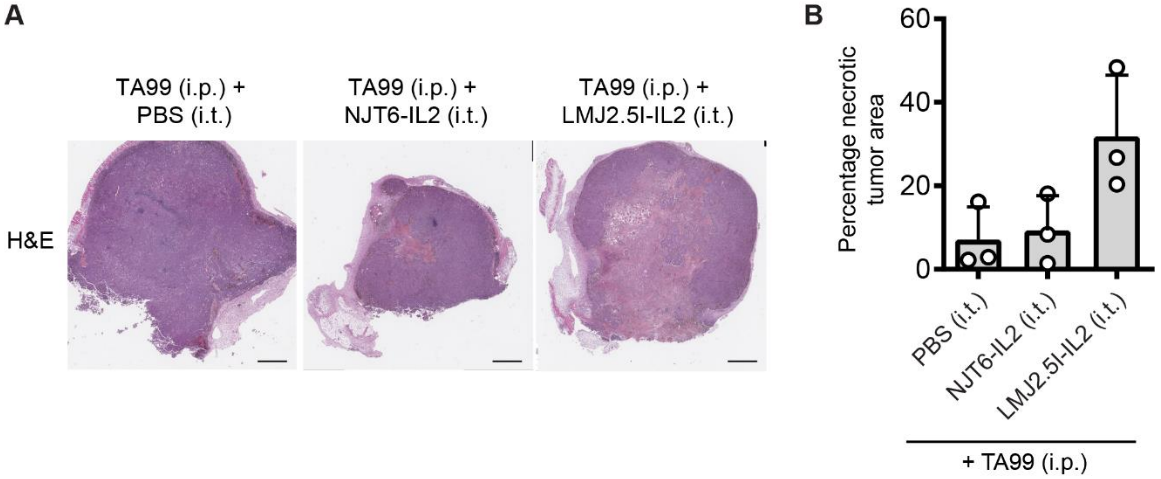

Fig. S9. Intratumoral administration of immunocytokines leads to necrosis. Mice were inoculated on day 0 with 1 M B16F10 cells subcutaneously in the right flank. Mice were treated with 100 μg TA99 (i.p.) on day 6, and PBS or 0.4 nmol nanobody-IL2 fusions (i.t.) on days 6 and 10. On day 12, tumors were excised for hematoxylin and eosin (H&E) staining. The LMJ2.5I-IL2 group had high levels of necrosis. (**A**), Representative H&E images of *n* = 3. Scale bar, 1000 μm. (**B**), Percentage of tumor area that is necrotic, calculated using ImageJ software (mean + SD, *n* = 3).

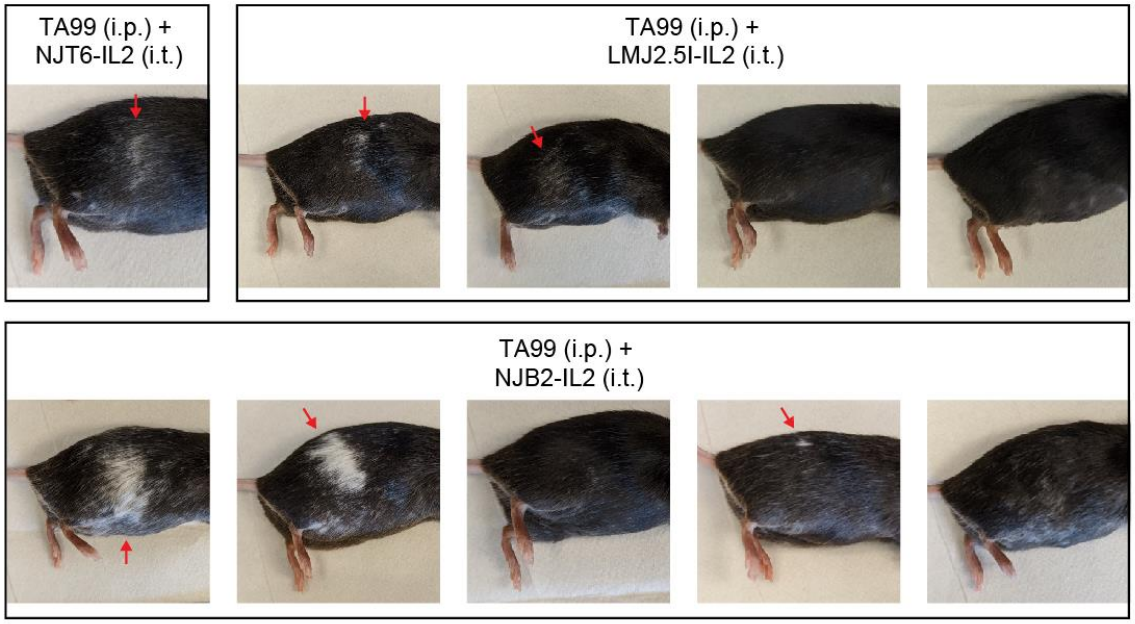

Fig. S10. Vitiligo response in mice treated with intratumoral immunocytokines. Mice were inoculated with 1 M B16F10 cells subcutaneously (s.c.) in the right flank on day 0. Mice were treated with 100 μg TA99 (i.p.) (days 6, 13, 20), and 0.4 nmol IL-2 fusions (i.t.) (days 6, 10, 13, 17, 20, 24). On day 94, all surviving mice were rechallenged with 0.1 M B16F10 cells s.c. in the left flank and tumor growth was monitored with no additional treatment. After an additional 6 to 9 months, mice that rejected rechallenge (*n* = 1 for TA99 + NJT6-IL2, *n* = 5 for TA99 + NJB2-IL2, *n* = 4 for TA99 + LMJ2.5I-IL2) were photographed on their right flank. Red arrows point towards the presence of vitiligo on six of the mice.

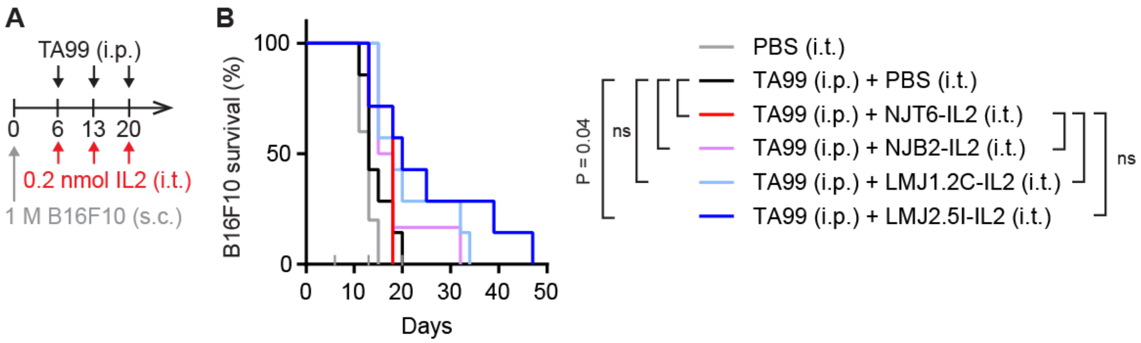

Fig. S11. Low dose intratumoral treatment of IL-2 fusions. (**A**), B16F10 study timeline. Mice were inoculated with 1 M B16F10 cells subcutaneously (s.c.) in the right flank on day 0. Mice were treated on indicated days with 100 µg TA99 (i.p.) and 0.2 nmol (6.4 µg) IL-2 fusions (i.t.). (**B**), Survival curves. Gray ticks above the x-axis mark treatment days. Survival comparisons were generated by a log-rank Mantel-Cox test. ns, not significant. n = 5 for PBS, n = 6~7 for all other groups.

Table S1. Amino acid sequences of nanobodies. Amino acid sequences are shown for recombinant nanobodies. The sequence for the nanobody is shown in blue. Glycine-serine linkers, sortase motifs (LPETG) and polyhistidine tags (HHHHHH) are shown in black.

**Nanobody**	**Sequence**
NJT6	QVQLVETGGDLVQPGGSLRLSCAASGLTLDYYAIGWVRQAPGKEREGVSCIT<br>PQDGNTYYDDSVMGRFTILRDNAKNMVYLQMNNLKPEDTAVYFCAAAGALTL<br>DPSEYEYWGQGTQVTVSSGGGSLPETGGHHHHHH
NJB2	QVQLVETGGGLVQAGGSLRLSCAASGSTFSHNAGGWYRQAPEKQRELVAGI<br>SSDGNINYADSVKDRFTISRDNASNTMYLQMNNLKPEDTAVYVCNIRGSYGN<br>TYYSRWGQGTQVTVSSGGGSLPETGGHHHHHH
LMJ1.2C	QVQLVETGGGLVQAGGSLRLSCAASGSTFSHSAGGWYRQAPEKQRELVAGI<br>RSDGNINYADSVKDRFTISRDNASNTMYLQMNNLKPEDTAVYVCNIRGSYGN<br>TYYSRWGQGTQVTVSSGGGSLPETGGHHHHHH
LMJ2.5I	RVQLVETGGGLVQAGGSLRLSCAVSGSTFSHSAGGWYRQAPEKQRELVAGI<br>RSDGNINYADSVKDRFTISRDNASNTMYLQMNNLKPEDTAVYVCNIRGSYGN<br>TYYSRWGQGAQVTVSSGGGSLPETGGHHHHHH
LMJ1.2G	QVQLVETGGGLVQAGGSLRLSCAASGSTFSHSAGGWYRQAPEKQRELVAGI<br>GSDGNINYADSVKDRFTISRDNASNTMYLQMNNLKPEDTAVYVCNIRGSYGN<br>TYYSRWGQGTQVTVSSGGGSLPETGGHHHHHH
LMJ1.3J	QVQLVGTGGGLVQAGGSLRLSCAASGSTFSHSAGGWYRQAPEKQRELVAGI<br>SSAGNINYADSVKDRFTISRDNASNTMYLQMNNLKPEDTAVYVCNIRGSYGNT<br>YYSRWGQGTQVTVSSGGGSLPETGGHHHHHH
LMJ2.5C	QVQLVETGGGLVQAGGSLRLSCAASGSTFSHSAGGWYRQAPEKQRELVAG<br>VRSDGNINYADSVKDRFTISRDNASNTMYLQMNNLKPEDTAVYVCNIRGSYG<br>NTYYSRWGQGIQVTVSSGGGSLPETGGHHHHHH
LMJ2.5G	QVQLVETEGGLVQAGGSLRLSCAASGSTFSHSAGGWYRQAPEKQRELVAGI<br>RSDGNINYADSVKDRFTISRDNASNTMYLQMNNLKPEDTAVYVCNIRGSYGN<br>TYYSRWGQGTQVTVSSGGGSLPETGGHHHHHH
LMJ2.5H	QVQLVETGGGLVQAGGSLRLSCAVSGSTFSHSAGGWYRQAPEKQRELVAGI<br>RSDGNINYADSVKDRFTISRDNASNTMYLQMNNLKPEDTAVYVCNIRGSYGN<br>TYYSRWGQGTQVTVSSGGGSLPETGGHHHHHH
LMJ2.5J	QVQLVETRGGLVQAGGSLRLSCAVSGSTFSHSAGGWYRQAPEKQRELVAGI<br>GSAGNINYADSVKDRFTISRDNASNTMYLQMNNLKPEDTAVYVCNIRGSYGN<br>TYYSRWGQGTQVTVSSGGGSLPETGGHHHHHH
LMJ2.4C	QVQLVEAGGGLVQAGGSLRLSCAASGSTFSHSAGGWYRQAPEKQRELVAGI<br>RSDGNINYADSVKDRFTISRDNASNTMYLQMNNLKPEDTAVYVCNIRGSYGN<br>TYYSRWGQGIQVTVSSGGGSLPETGGHHHHHH
LMJ2.6A	RVQLVETGGGLVQAGGSLRLSCAVSGRTFSHSAGGWYRQAPEKQRELVAGI<br>RSDGNINYADSVKDRFTISRDNASNTMYLQMNNLKPGDTAVYVCNIRGSYGN<br>TYYSRWGQGTQVTVSSGGGSLPETGGHHHHHH

Table S2. Amino acid sequences of nanobody-IL-2 fusions. Amino acid sequences are shown for nanobody-IL-2 fusions. The sequence for the nanobody is shown in blue and the sequence for murine IL-2 is shown in red. Glycine-serine linkers, sortase motifs (LPETG), and polyhistidine tags (HHHHHH) are shown in black.

IL-2 fusion	Sequence
NJT6-IL2	QVQLVETGGDLVQPGGSLRLSCAASGLTLDYYAIGWVRQAPGKEREGVSCI<br>TPQDGNTYYDDSVMGRFTILRDNAKNMVYLQMNNLKPEDTAVYFCAAAGAL<br>TLDPSEYEYWGQGTQVTVSSGGGGGSAPTSSSTSSSTAEAQQQQQQQQQ<br>QQQHLEQLLMDLQELLSRMENYRNLKLPRMLTFKFYLPKQATELKDLQCLED<br>ELGPLRHVLDLTQSKSFQLEDAENFISNIRVTVVKLKGSDNTFECQFDDESAT<br>VVDFLRRWIAFCQSIISTSPQLPETGGHHHHHH
NJB2-IL2	QVQLVETGGGLVQAGGSLRLSCAASGSTFSHNAGGWYRQAPEKQRELVAG<br>ISSDGNINYADSVKDRFTISRDNASNTMYLQMNNLKPEDTAVYVCNIRGSYG<br>NTYYSRWGQGTQVTVSSGGGGGSAPTSSSTSSSTAEAQQQQQQQQQQQ<br>QHLEQLLMDLQELLSRMENYRNLKLPRMLTFKFYLPKQATELKDLQCLEDEL<br>GPLRHVLDLTQSKSFQLEDAENFISNIRVTVVKLKGSDNTFECQFDDESATVV<br>DFLRRWIAFCQSIISTSPQLPETGGHHHHHH
LMJ1.2C-IL2	QVQLVETGGGLVQAGGSLRLSCAASGSTFSHSAGGWYRQAPEKQRELVAG<br>IRSDGNINYADSVKDRFTISRDNASNTMYLQMNNLKPEDTAVYVCNIRGSYG<br>NTYYSRWGQGTQVTVSSGGGGGSAPTSSSTSSSTAEAQQQQQQQQQQQ<br>QHLEQLLMDLQELLSRMENYRNLKLPRMLTFKFYLPKQATELKDLQCLEDEL<br>GPLRHVLDLTQSKSFQLEDAENFISNIRVTVVKLKGSDNTFECQFDDESATVV<br>DFLRRWIAFCQSIISTSPQLPETGGHHHHHH
LMJ2.5I-IL2	RVQLVETGGGLVQAGGSLRLSCAVSGSTFSHSAGGWYRQAPEKQRELVAG<br>IRSDGNINYADSVKDRFTISRDNASNTMYLQMNNLKPEDTAVYVCNIRGSYG<br>NTYYSRWGQGAQVTVSSGGGGGSAPTSSSTSSSTAEAQQQQQQQQQQQ<br>QHLEQLLMDLQELLSRMENYRNLKLPRMLTFKFYLPKQATELKDLQCLEDEL<br>GPLRHVLDLTQSKSFQLEDAENFISNIRVTVVKLKGSDNTFECQFDDESATVV<br>DFLRRWIAFCQSIISTSPQLPETGGHHHHHH
NJB2-IL2-mt	QVQLVETGGGLVQAGGSLRLSCAASGSTFSHNAGGWYRQAPEKQRELVAG<br>ISSDGNINYADSVKDRFTISRDNASNTMYLQMNNLKPEDTAVYVCNIRGSYG<br>NTYYSRWGQGTQVTVSSGGGGGSAPTSSSTSSSTAEAQQQQQQQQQQQ<br>QHLEQLLMDLQELLSRMENYRNLKLPDMLTFEFYLPKQATELKDLQCLEREL<br>GPLRHVLDLTQSKSFQLEDAENFISNIRRTVVKLKGSDNTFECQFDDESATVV<br>DFLRRWIAFCTSIISTSPQLPETGGHHHHHH
